# Chemical Knockdown of Phosphorylated p38 Mitogen-Activated Protein Kinase (MAPK) as a Novel Approach for the Treatment of Alzheimer′s Disease

**DOI:** 10.1021/acscentsci.2c01369

**Published:** 2023-03-01

**Authors:** Seung Hwan Son, Na-Rae Lee, Min Sung Gee, Chae Won Song, Soo Jin Lee, Sang-Kyung Lee, Yoonji Lee, Hee Jin Kim, Jong Kil Lee, Kyung-Soo Inn, Nam-Jung Kim

**Affiliations:** † College of Pharmacy, Kyung Hee University, Seoul 02447, Republic of Korea; ‡ Prazer Therapeutics Inc., Beobwon-ro 9-gil 26, Songpa-gu, Seoul 05836, Republic of Korea; § Department of Bioengineering and Institute of Nanoscience and Technology, Hanyang University, Seoul 04763, Republic of Korea; ∥ College of Pharmacy, Chung-Ang University, Seoul, 06974, Republic of Korea

## Abstract

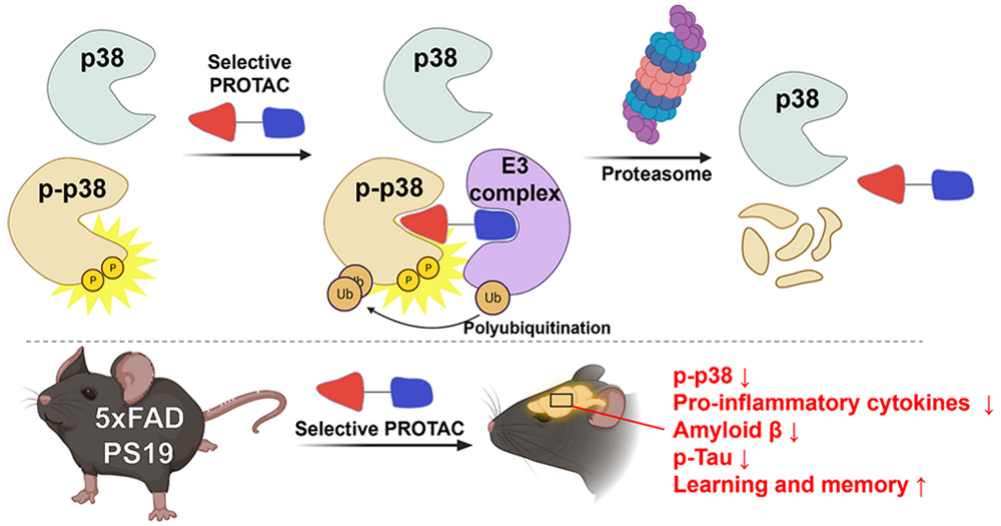

Targeted protein degradation (TPD) provides unique advantages over gene knockdown in that it can induce selective degradation of disease-associated proteins attributed to pathological mutations or aberrant post-translational modifications (PTMs). Herein, we report a protein degrader, PRZ-18002, that selectively binds to an active form of p38 MAPK. PRZ-18002 induces degradation of phosphorylated p38 MAPK (p-p38) and a phosphomimetic mutant of p38 MAPK in a proteasome-dependent manner. Given that the activation of p38 MAPK plays pivotal roles in the pathophysiology of Alzheimer’s disease (AD), selective degradation of p-p38 may provide an attractive therapeutic option for the treatment of AD. In the 5xFAD transgenic mice model of AD, intranasal treatment of PRZ-18002 reduces p-p38 levels and alleviates microglia activation and amyloid beta (Aβ) deposition, leading to subsequent improvement of spatial learning and memory. Collectively, our findings suggest that PRZ-18002 ameliorates AD pathophysiology via selective degradation of p-p38, highlighting a novel therapeutic TPD modality that targets a specific PTM to induce selective degradation of neurodegenerative disease-associated protein.

## Abstract 2

Selective degradation of phosphorylated p38 MAPK was developed and evaluated for its biological effect on the treatment of Alzheimer’s disease in the 5xFAD and PS19 transgenic mouse model.

## Introduction

Recent progress in the field of targeted protein degradation (TPD) has proven its immense potential as a novel therapeutic modality in drug discovery. In 2015, Bradner and his colleagues devised a phthalimide-based small molecule that promotes degradation of transcriptional coactivator BRD4 by hijacking the Cereblon (CRBN) E3 ubiquitin ligase complex.^1^ In the same year, Crews and his colleagues also reported a TPD technology recruiting the von Hippel–Lindau (VHL) E3 ligase complex, commonly referred to as proteolysis-targeting chimeras (PROTACs).^2^ These technologies feature bifunctional small molecules that bring the proteins of interest into proximity with the E3 ubiquitin ligase complexes for ubiquitination and subsequent proteasomal degradation.^3^ Such TPD-based small molecules have several advantages over traditional small molecule inhibitors in that they eliminate the target protein instead of modulating its function.^4^ TPD technology thus can complement nucleic acid-based gene knockdown in removing unwanted intracellular proteins. In addition, the TPD technique can target a plethora of proteins in various compartments of the cell, including disease-causing proteins that have previously been considered undruggable with the conventional small-molecule approach. Recently, several strategies have been suggested to potentiate therapeutic efficacy of TPD technology.^4^ In particular, TPD molecules that recognize and bind to the protein with specific post-translational modifications (PTMs), such as phosphorylation, may be a novel strategy to induce selective degradation of pathological proteins attributed to aberrant PTMs.^5^ However, a TPD molecule specifically targeting post-translationally modified proteins has not been reported yet.

Mitogen-activated protein kinases (MAPKs) belong to a family of serine/threonine protein kinases that participate in signaling pathways regulating cellular functions in response to various extracellular stimuli.^6^ Among three MAPKs established in mammalian cells—extracellular signal-regulated kinase (ERK), *N*-terminal kinase (JNK), and p38—p38 has been considered to be activated mainly by pro-inflammatory cytokines and environmental stresses.^7^ Accumulation of p-p38 through the kinase cascade is one of pathological hallmarks. It has been reported that phosphorylated p38 (p-p38) is significantly upregulated under pathological conditions, such as chronic inflammation, thus triggering downstream signal transduction and leading to pathological deterioration.^8^ P38 dysfunction has been implicated in a variety of medical disorders, such as neuroinflammation, ischemia, and cognitive impairment.^9−12^ Our previous study showed that enzymatic inhibition of p38 alleviated pathological symptoms of Alzheimer′s disease (AD), particularly neuroinflammation and accumulation of beta-amyloid (Aβ) and tau proteins.^13^ The therapeutic potential of inhibiting p38 in neurodegeneration has been investigated in several clinical trials, but there has been no success yet partly due to off-target effects and insufficient efficacy.^14^

In this study, we use targeted protein degradation as a strategy to induce selective degradation of p-p38. Based on the phosphorylation-dependent conformational difference in p38, the glycine flip and DFG motif change, we rationally designed and synthesized a series of p-p38-degrading small molecules, consisting of a p-p38 ligand and pomalidomide that can recruit the CRBN E3 ubiquitin ligase complex. Among them, we found that compound **2**, namely PRZ-18002, displayed excellent kinase selectivity and efficiently degraded p-p38 as well as the constitutively active phosphomimetic mutant of p38 in several types of cells. The modeling study showed that PRZ-18002 nicely binds to the DFG-in conformation of p-p38 and gives adequate proximity to CRBN with the optimized linker moiety. Using a positioning device that allows us to circumvent the blood–brain barrier (BBB),^15^ we were able to administer PRZ-18002 intranasally into the brain of 5xFAD transgenic mice that recapitulated major features of AD. Importantly, we found that PRZ-18002 induced selective in vivo degradation of p-p38 and ameliorated neurodegenerative symptoms including neuroinflammation, Aβ deposition, and memory loss. Overall, this study highlights selective targeting of p38 bearing a specific PTM for proteasomal degradation, providing a novel therapeutic approach for the treatment of AD.

## Results

### Synthesis of p-p38 Degraders

**Scheme 1:**
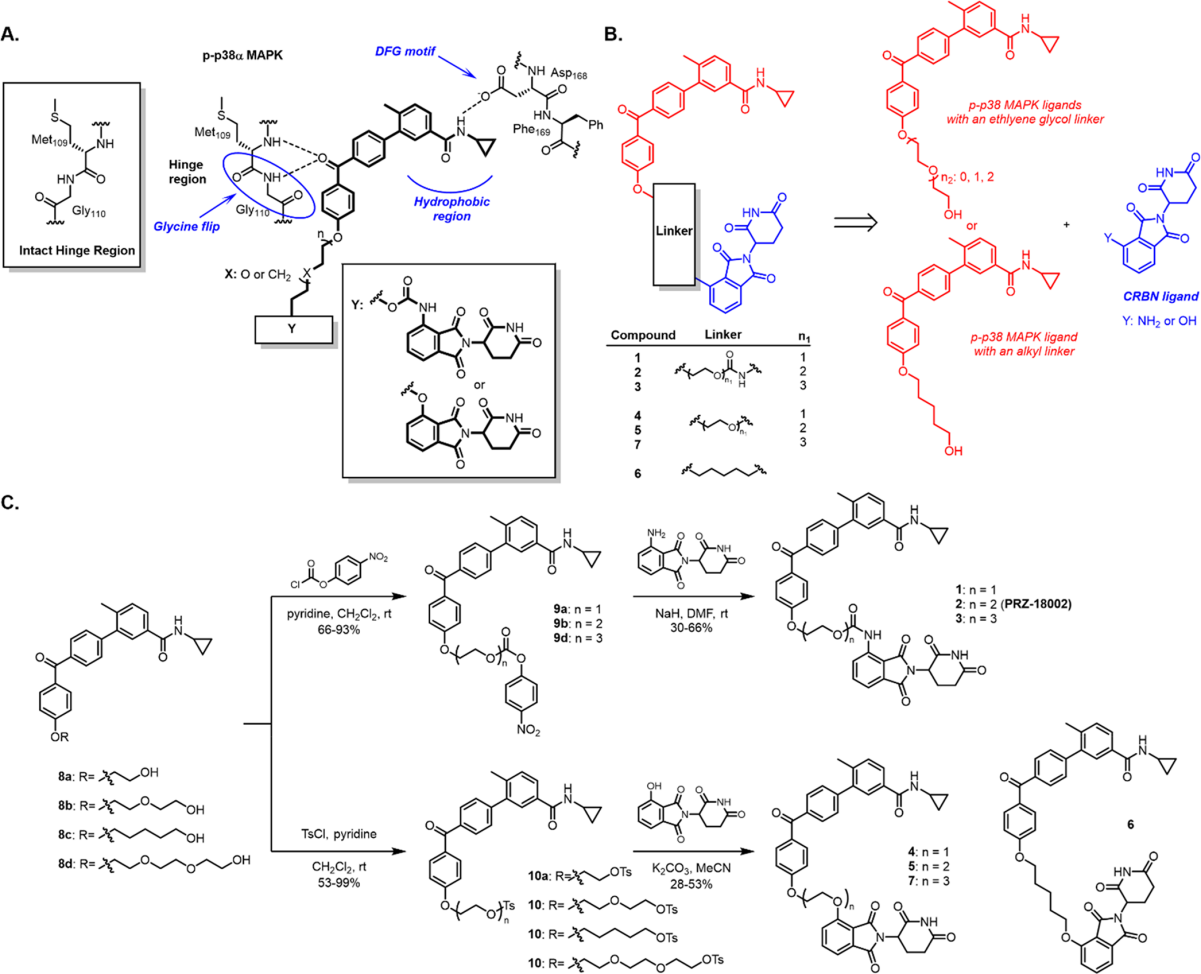
Selective p-p38 Degraders Are Designed and Synthesized on the Basis of Targeting Glycine Flip in the Hinge Region of p-p38 Active Sites: (A) Structures of Designed Degraders and Their Plausible Binding Modes. (B) Retrosynthetic Scheme of the Designed p-p38 Degraders. (C) Synthetic Scheme of Compounds **1**–**7**

In our previous study, we reported a series of novel p38 inhibitors that target the hinge region of p38.^16^ The benzophenone moiety of p38 inhibitor can form double hydrogen bonds with the NH groups of Gly110 and its adjacent linker residue Met109 at the hinge region of p38. This interaction occurs by inducing a glycine flip, in which a glycine residue undergoes rotation and replaces the position of the carbonyl oxygen with the NH group. While the amide proton of Gly110 is oriented away from the ATP-binding pocket of p38, the hydrogen bond acceptor of the inhibitor induces the glycine flip that directs the amide proton inward, leading to tight and selective binding with p38 (Scheme 1A). This flip interaction imposes high selectivity of the inhibitors to p38 because such an arrangement exists only in 9.2% of the kinome.^17^ Interestingly, it has been reported that the intrinsic conformation of phosphorylated p38 is analogous to the structure where the flip was induced by the p38 inhibitors,^18^ which allowed us to hypothesize that p-p38 might be a more favorable target of the inhibitors compared with their nonphosphorylated counterpart. Accordingly, in search of potent and selective degraders of phosphorylated p38, we designed and synthesized a series of chemical compounds (**1**–**7**) by conjugating the p38 inhibitor targeting a glycine flip^13^ and pomalidomide using linkers (Scheme 1B–C and Figure S1A).

### Identification of PRZ-18002 as a Selective Degrader of p-p38

**Figure 1:**
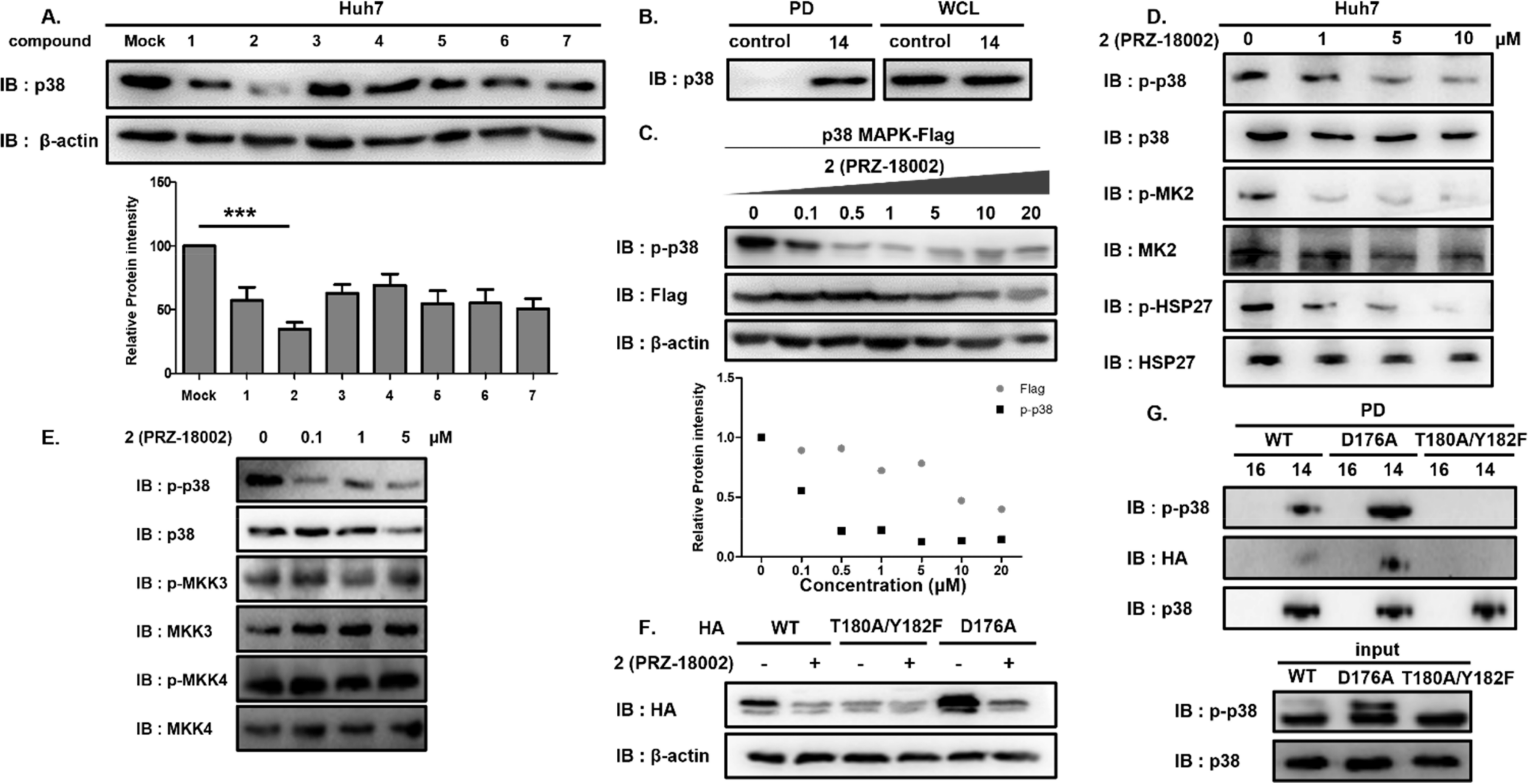
PRZ-18002 induces degradation of phospho-p38 protein in a proteasome-dependent manner. (A) Synthesized compounds were screened and PRZ-18002 successfully degrades p38. Huh7 cells were treated with p38 degraders at 10 μM for 24 h, and cell lysates were analyzed by immunoblotting using anti-p38 antibody and anti-β actin antibody. The bottom graph shows quantification of p38 levels. The immunoblot is representative of three independent experiments (*n* = 3). Data are shown as mean ± SEM (***P < 0.001). *P* values were calculated with analysis of variance (ANOVA). (B) p38 ligand part in **2** binds p38. Biotin-conjugate (**14**) of the p38 ligand part in PRZ-18002 and biotin-conjugate of benzene (**16**, control compound) were manufactured to confirm binding with PRZ-18002 and p38. Lysates of BV-2 cells were incubated with 20 μM **14** or **16** at 37 °C for 2 h and then subjected to a pulldown assay. The resulting precipitates and whole cell lysates were analyzed by immunoblotting using p38 antibody. (C) PRZ-18002 preferentially degrades p-p38 over p38 in a concentration-dependent manner. HEK293T cells expressing Flag-tagged p38 were incubated with PRZ-18002 for 24 h at the indicated concentration. Cell lysates were analyzed by immunoblotting with indicated antibodies. (D) PRZ-18002 treatment results in suppression of p-pMK2 and p-HSP27 levels. Huh7 cells were treated with PRZ-18002 for 24 h at the indicated concentrations, and their lysates were analyzed by immunoblotting using p-pMK2 and p-HSP27 antibodies. (E) p-MKK3 and p-MKK3 levels are not decreased by **2**, unlike p-p38. Huh7 cells were incubated with PRZ-18002 for 24 h at the indicated concentrations. Cell lysates were analyzed by immunoblotting. (F) PRZ-18002 preferentially interacts with the active form of p38. HEK293T cells expressing p38 wild-type, p38 inactive form (D180A/Y182F mutant) or p38 active form (D176A mutant) were treated with PRZ-18002 for 24 h and then were analyzed by immunoblotting. (G) p38 ligand part in PRZ-18002 selectively and preferentially binds to a phosphomimetic mutant of p38. HEK293T cells were transfected with p38 wild-type, p38 inactive form, or p38 active form followed by incubation for 24 h. Cell lysates were incubated with **14** or **16** at 4 °C for 24 h. Pulldown assays were conducted using streptavidin beads, and then the p-p38 protein level and p38 protein level were determined by immunoblotting.

**Figure 2:**
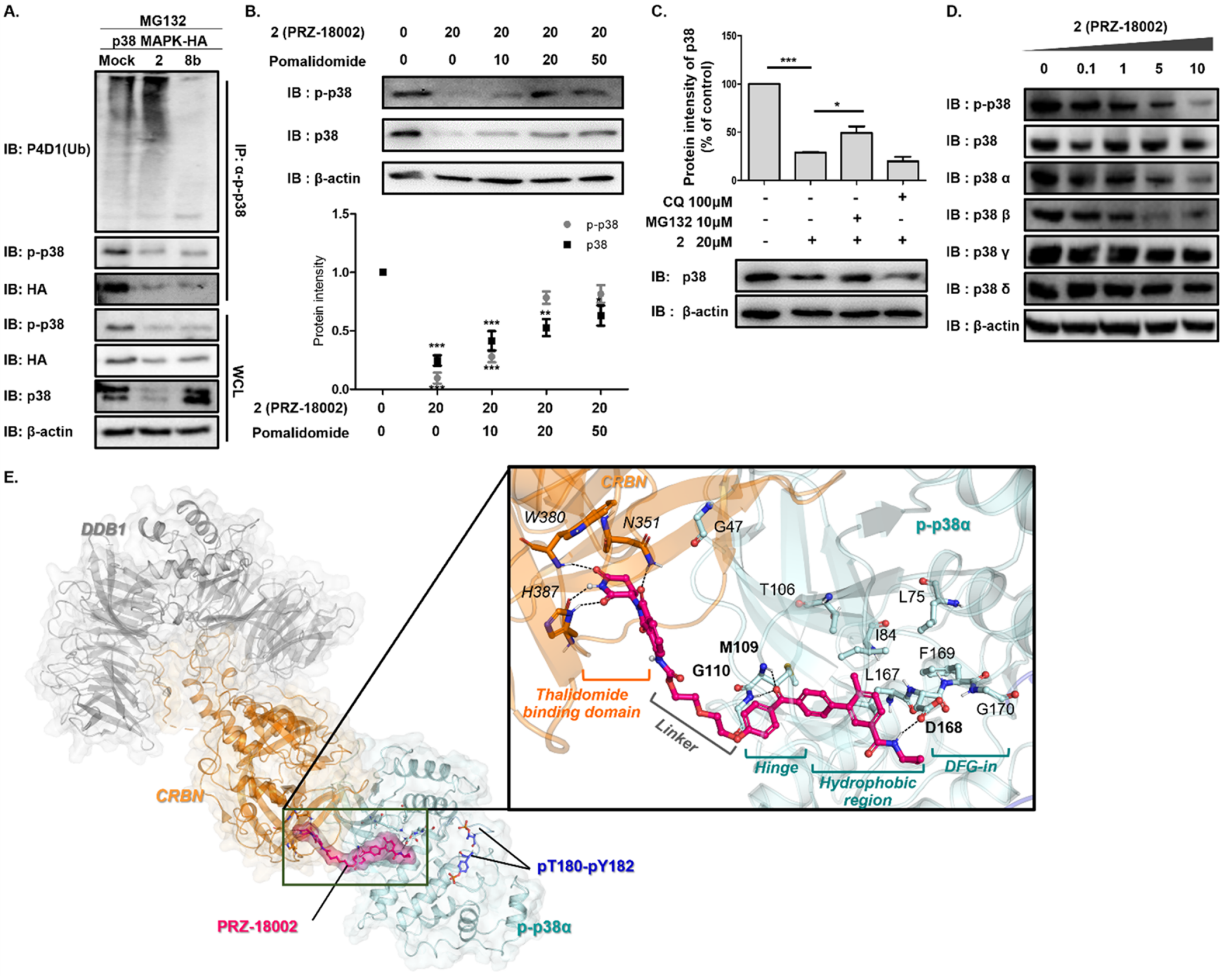
PRZ-18002 induces degradation of p-p38 and p38 protein in a proteasome-dependent manner. (A) PRZ-18002 induces ubiquitination of p-p38. HEK293T cells were transfected with p38-HA plasmid and incubated for 24 h. HEK293T cells overexpressing p38 were treated for 24 h with either compound [10 μM PRZ-18002 or 10 μM **8b**] or DMSO together with 5 μM MG132. An immunoprecipitation assay for each sample was performed, and the result was analyzed by immunoblotting. (B) In Huh7 cells, PRZ-18002-mediated degradation of p-p38 and p38 was significantly hindered in the presence of pomalidomide. Immunoblot analysis for p-p38 and p38 was performed after 6 h pretreatment with either DMSO or pomalidomide, followed by treatment with **2** for 24 h. Cell lysates were analyzed by immunoblotting. The immunoblot is representative of three independent experiments (*n* = 3). Data are shown as mean ± SEM (*P < 0.05; **P < 0.01; ***P < 0.001 vs control group). (C) p-p38 is degraded by PRZ-18002 in a proteasome-dependent manner. Huh7 cells were treated with DMSO or 20 μM PRZ-18002 together with 100 μM CQ or 10 μM MG132. Cell lysates were analyzed with anti-p38 and anti-β actin antibodies. The upper graph shows quantification of p38 levels. The immunoblot is representative of three independent experiments (*n* = 3). Data are shown as mean ± SEM (*P < 0.05; ***P < 0.001). (D) p-p38 degradation by PRZ-18002 has selectivity for p38 alpha and p38 beta rather than p38 gamma and p38 delta. Huh7 cells were treated with p38 degrader at 0.1, 1, 5, 10 μM for 24 h. Cell lysates were analyzed with antibodies as indicated. (E) Predicted binding mode of PRZ-18002 in the active p38α and E3 ligase complex. The structures of the p-p38α, CRBN, and DNA damage binding protein (DDB1) in E3 ligase complex are colored in cyan, orange, and gray, respectively. The phosphorylated T180 and Y182 residues in the active p38α are marked as sticks with the carbon atoms in blue. The bound PRZ-18002 molecule is represented as sticks with the carbon atom in magenta. The interacting residues in p-p38α and CRBN are also depicted as sticks with their residue numbers marked.

When we tested the ability of compounds (**1**–**7**) to reduce the level of p38 protein in Huh7 cells, **2** (PRZ-18002) was shown to be the most effective degrader of p38 (Figure 1A). We also synthesized compound **14**, biotin-conjugate of p38 ligand part in **2** (Figure S1B), and confirmed a robust interaction between compound **14** and p38 by a pulldown assay (Figure 1B), indicating that the p38 ligand in PRZ-18002 is responsible for the interaction with p38. In the presence of PRZ-18002, significant reduction of p-p38 was observed in a concentration-dependent manner, with a half-maximal degradation concentration (DC_50_) of 93 nM (Figure 1C), compared with Flag-tagged p38 (DC_50_ of 7309 nM), indicating higher selectivity of PRZ-18002 toward activated p-p38 compared with inactive p38 in the nonphosphorylated state. The docking study showed that the p38 ligand part of PRZ-18002 would nicely bind to the hinge region of the phosphorylated p38α DFG-in conformation via double hydrogen bonding by glycine flip (Figure S2A, note that a more detailed interaction is shown in Figure 2E), while the compound might have a steric clash at the binding site of DFG-out (i.e., inactive) conformation (Figure S2B). PRZ-18002 also decreased the phosphorylation level of downstream targets of p38, MK2, and HSP27, in a concentration-dependent manner (Figure 1D). In addition, PRZ-18002 showed excellent kinase selectivity against a panel of 96 different kinases (Figure S3), which are considered to be functionally or structurally similar to p38 based on the kinome analysis and the cross reactivity analysis with other p38 inhibitors such as SB-203580, BIRB-796, and Neflamapimod (VX-745).^19^ We also observed a substantial reduction in the protein level of p-p38 compared with other MAPKs, such as ERK, JNK, and MEK, in their phosphorylated forms (Figure S4). In addition, the phosphorylation status of MKK3 and MKK4 kinases, upstream MAPK kinases (MAPKKs) responsible for the phosphorylation of p38, did not show any significant change upon treatment with PRZ-18002 (Figure 1E).

Collectively, these results indicated that PRZ-18002 selectively targets and reduces the level of activated p38 without affecting MAPKs or MAPKKs. We further verified the preference of PRZ-18002 on activated p38 using its constitutively active phosphomimetic mutant (D176A) and inactive mutant (T180A/Y182F).^20^ Treatment with PRZ-18002 resulted in a significant reduction of active p38 mutant (D176A) in preference to wild-type p38, whereas it showed a marginal effect on inactive p38 mutant (T180A/Y182F) (Figure 1F). In addition, a pulldown assay using compound **14**, biotin-conjugate of p38 ligand part in **2**, showed that the p-p38 ligand of PRZ-18002 selectively and preferentially binds to a phosphomimetic mutant of p38 (Figure 1G). When we proceeded to test if PRZ-18002 induces ubiquitination of p-p38 for subsequent degradation, we observed robust ubiquitination of p-p38, whereas compound **8b**, a p38 ligand lacking pomalidomide, failed to do so (Figure 2A). PRZ-18002-mediated degradation of p-p38 and p38 was significantly hindered in the presence of pomalidomide, a CRBN binder, indicating that the E3 ubiquitin ligase complex consisting of CRBN is involved in the targeted degradation of p-p38 and p38 by PRZ-18002 (Figure 2B). The effect of PRZ-18002 on degradation of p38 was diminished upon treatment with the proteasomal inhibitor MG132, while it remained unaffected by the lysosomal inhibitor chloroquine (CQ), indicating that PRZ-18002-mediated degradation of p38 occurs via a ubiquitin-proteasome system (UPS). (Figure 2C). In addition, reduction of p38 by PRZ-18002 has selectivity for p38 alpha and beta rather than p38 gamma and delta (Figure 2D). In addition, compared with a p38 inhibitor, it was confirmed that the p-p38 degradation by p38 degradation ability of the p38 degrader persisted for a long time even after treatment and washing (Figure S5B). Taken together, these data demonstrate that PRZ-18002 selectively and preferentially binds to the active form of p38 and induces ubiquitination and subsequent degradation of p-p38 in a proteasome-dependent manner.

To get structural insights of the mode of action for PRZ-18002, a molecular modeling study was conducted. The p38 ligand part and the *S*-pomalidomide moiety of PRZ-18002 were separately docked to the X-ray crystal structures of p-p38α (PDB id: 6ZQS)^21^ and CRBN (PDB id: 5FQD),^22^ respectively. By using the geometry of the kinase domain of casein kinase 1 bound to the E3 ligase complex (PDB id: 5FQD),^22^ the ternary complex model of the p38 kinase domain, CRBN, DNA damage binding protein 1 (DDB1) of the E3 ligase complex were constructed. After the linker moiety was manually modeled to connect the p38 ligand and pomalidomide part, the quaternary complex of PRZ-18002, p-p38α, CRBN, and DDB1 was optimized based on energy minimization. The final structural model showed that the linker moiety of PRZ-18002 provides good proximity between the thalidomide binding domain of CRBN and the hinge region of the p38 kinase domain (Figure 2E). Especially, the benzophenone moiety interacts with M109 and G110 via double H-bonds (glycine flip), maintaining the linker’s direction toward CRBN. In addition to the glycine flip, the binding mode also explains the compound’s preference to the phosphorylated p38 due to the interaction with the DFG-in motif (Figure S2).

### Suppression of p38-Mediated Neuroinflammation by PRZ-18002

**Figure 3:**
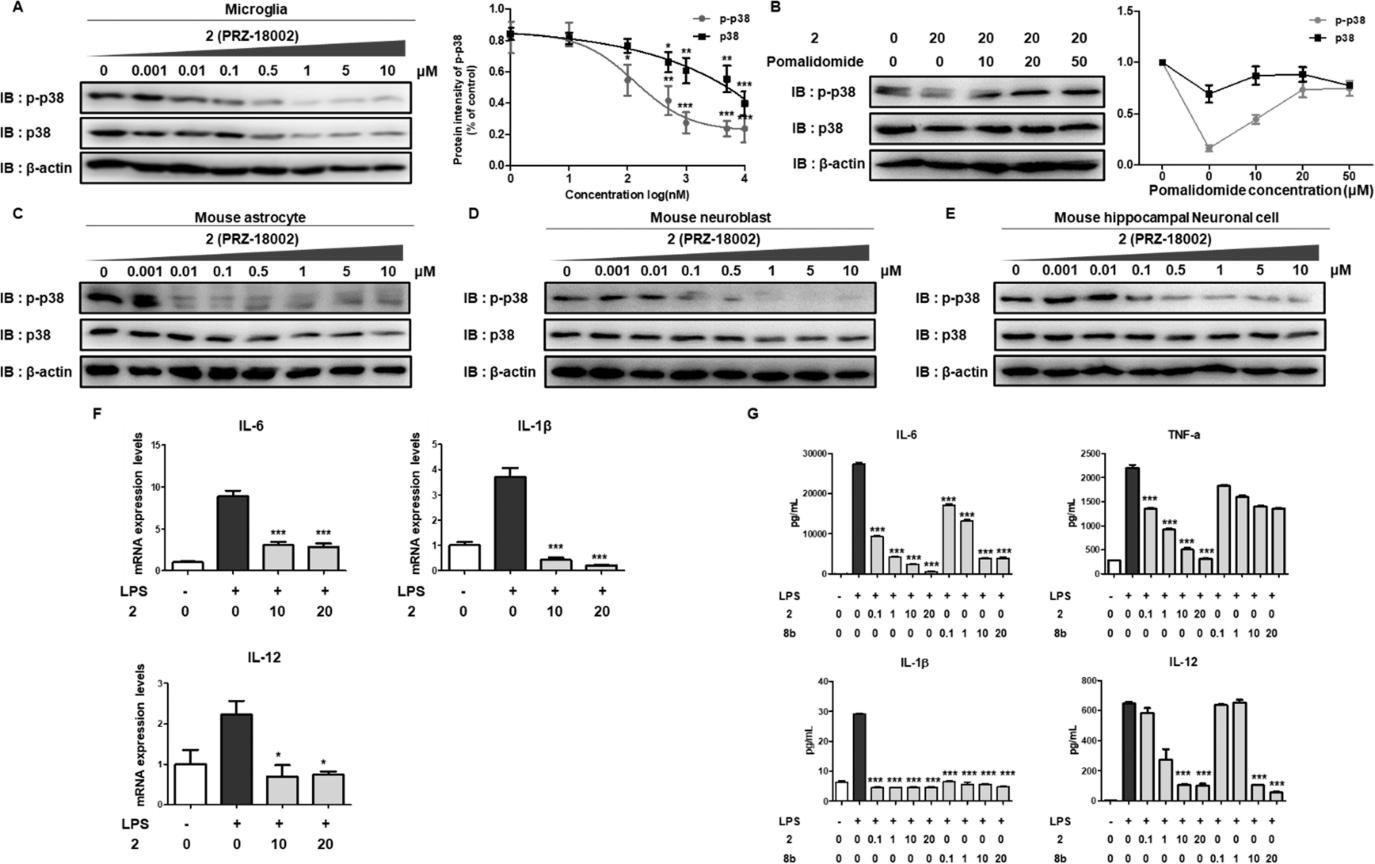
PRZ-18002 leads to decreased pro-inflammation production following degradation of both p-p38 and p38 protein in diverse brain cells. (A) PRZ-18002 induces dose-dependent decrease of p-p38 and p38. BV-2 cells, mouse microglial cells, were treated for 24 h with increasing doses of PRZ-18002, and both p-p38 and p38 levels were analyzed by immunoblotting. Right panel shows the quantification of immunoblotting. The immunoblot is representative of three independent experiments (*n* = 3). Data are shown as mean ± SEM (*P < 0.05; **P < 0.01; ***P < 0.001 vs control group). (B) In BV2 cells, PRZ-18002-mediated degradation of p-p38 and p38 was significantly hindered in the presence of pomalidomide. Cells were treated for 24 h with 20 μM PRZ-18002 in the absence or presence of pomalidomide. (C–E) p-p38 and p38 levels upon treatment with PRZ-18002 were significantly decreased in mouse astrocyte (CD-D1a) (C), mouse neuroblast (N2a) (D), and mouse hippocampal neuronal cells (HT22) (E). (F) mRNA levels of IL-6, IL-1β, and IL-12 are lowered by PRZ-18002 treatment. After 16 h of PRZ-18002 treatment (0–20 μM), BV-2 cells were stimulated with 1 μg/mL LPS for 8 h. The mRNA level of each sample was determined by qPCR analysis. Data are shown as mean ± SEM (*P < 0.05; ***P < 0.001 vs LPS-only group). (G) Treatment with PRZ-18002 results in suppression of IL-6, IL-1β, IL-12, and TNFα levels. After 24 h of PRZ-18002 or 8b treatment (0–20 μM), BV-2 cells were stimulated with 1 μg/mL LPS for 24 h. The amount of each protein was measured using a cytokine Luminex assay (upper graph) and ELISA (bottom graph). The assay was performed in quadruplicate (*n* = 4). Data are shown as mean ± SEM (*P < 0.05; **P < 0.01; ***P < 0.001 vs LPS-only group).

In order to evaluate the potential effect of PRZ-18002 in impeding neuroinflammation, we examined if PRZ-18002 was capable of suppressing proinflammatory responses in the brain. First, in BV-2 microglial cells, we confirmed that PRZ-18002 substantially decreased p-p38 in a dose-dependent manner (Figure 3A). Consistent with previous observations in Huh7 cells (Figure 2B), PRZ-18002-mediated degradation of p-p38 in BV-2 cells was hampered in the presence of pomalidomide, suggesting that the protein degradation activity of PRZ-18002 depends on the interaction with CRBN (Figure 3B). Furthermore, PRZ-18002 was shown to have a potent degradation activity for p-p38 over p38 in various neuronal cells, including mouse astrocytes (C8-D1A), mouse neuroblasts (N2a), and mouse hippocampal neuronal cells (HT22) (Figure 3C–E). In order to ensure PRZ-18002-induced degradation of p-p38 as a regulator of proinflammatory cytokines, we measured the mRNA expression profile of proinflammatory cytokines downstream of p38. While the mRNA levels of proinflammation cytokines such as IL-6, IL-1β, and IL-12 were markedly decreased (Figure 3F), the mRNA level of p38 remained unaffected upon treatment with PRZ-18002 (Figure S6A). In addition, we observed that production of proinflammatory cytokines, IL-6, IL-1β, IL-12, and TNF-α, was notably reduced in the presence of PRZ-18002 compared to compound **8b** (Figure 3G). While treatment with the protein translation inhibitor cycloheximide (CHX) had no effect on the level of p-p38 and p38, cotreatment with PRZ-18002 markedly decreased the level of p-p38 (Figure S6B), suggesting that reduced level of p-p38 by PRZ-18002 does not occur from inhibiting the protein synthesis of p38.

### Alleviation of AD Pathologies by PRZ-18002 in the 5xFAD Mice

**Figure 4:**
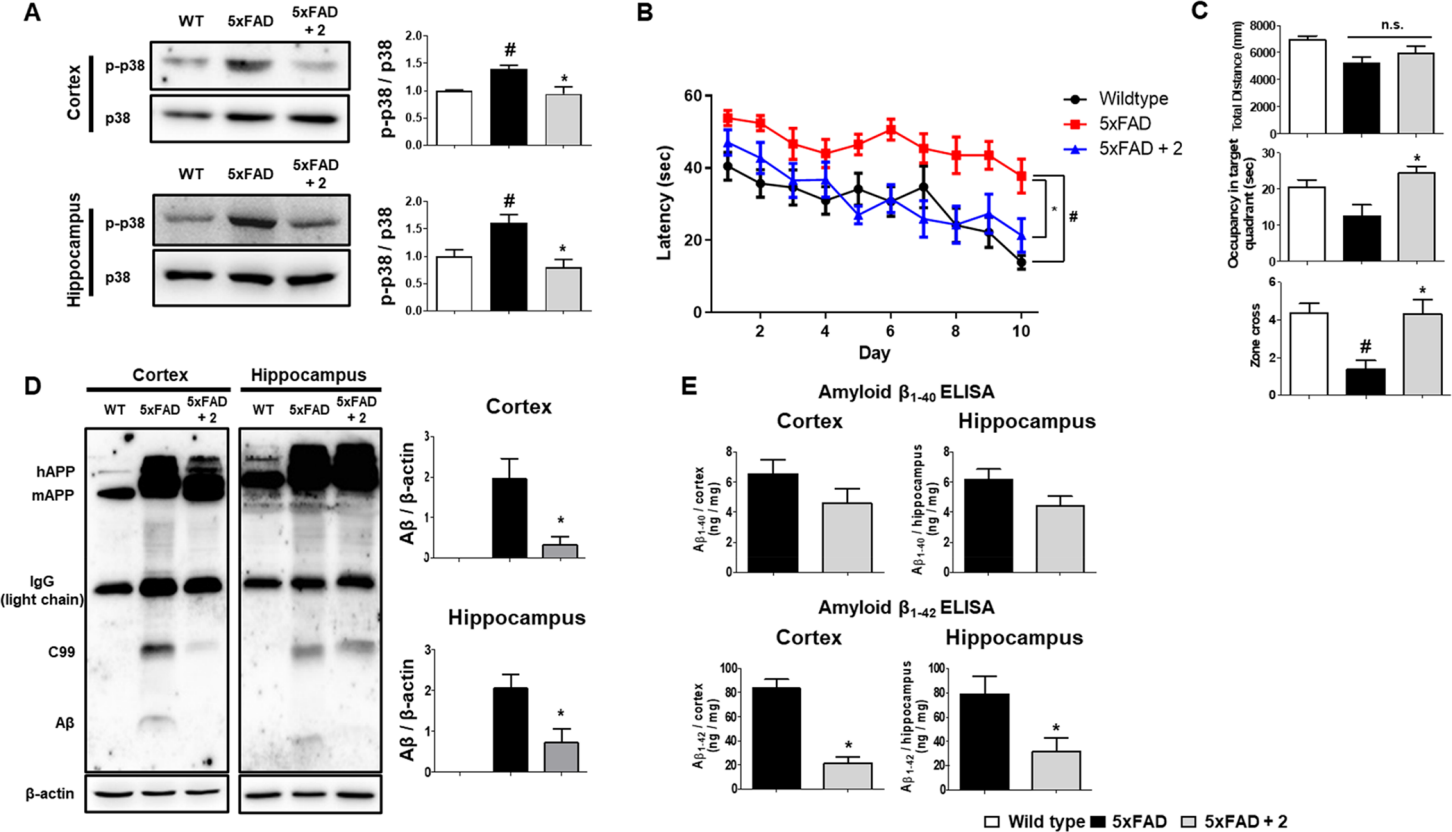
PRZ-18002 treatment diminished the phospho-p38 level and improved AD pathologies in 9-month-old 5xFAD mice. (A) Brain p-p38 levels of the mice. PRZ-18002 treated group showed significantly decreased p-p38 levels in brain, compared to the vehicle-treated 5xFAD group. Data are shown as mean ± SEM (WT; *n* = 4, 5xFAD; *n* = 5, 5xFAD+**2**; *n* = 5, one-way ANOVA, ^#^*P* < 0.05 vs wild-type, *P < 0.05 vs vehicle-treated 5xFAD). (B) Morris water maze latency during the training period. The 5xFAD group took longer to arrive at the hidden platform than the wild-type group, while the PRZ-18002 treated 5xFAD group showed significantly improved performance. Data are shown as mean ± SEM (WT; *n* = 11, 5xFAD; *n* = 10, 5xFAD+**2**; *n* = 10, GEE analysis, ^#^*P* < 0.05 vs wild-type, *P < 0.05 vs vehicle-treated 5xFAD). (C) Tracing analysis during probe task. Vehicle treated and PRZ-18002 treated 5xFAD group showed no significant difference in total swimming distance. Meanwhile, time spent in target quadrant and zone cross number were significantly improved than vehicle-treated 5xFAD group. Data are shown as mean ± SEM (WT; *n* = 11, 5xFAD; *n* = 10, 5xFAD+**2**; *n* = 10, one-way ANOVA, ^#^*P* < 0.05 vs wild-type, *P < 0.05 vs vehicle-treated 5xFAD). (D, E) Protein levels of brain Aβ were assessed by immunoblotting (D) and Aβ ELISA (E) in the mice. PRZ-18002 treated 5xFAD mice showed significantly decreased Aβ levels in brain, compared to vehicle-treated 5xFAD mice. Data are shown as mean ± SEM (WT; *n* = 4, 5xFAD; *n* = 5, 5xFAD+**2**; *n* = 5, Student’s *t* test, *P < 0.05 vs vehicle-treated 5xFAD).

**Figure 5:**
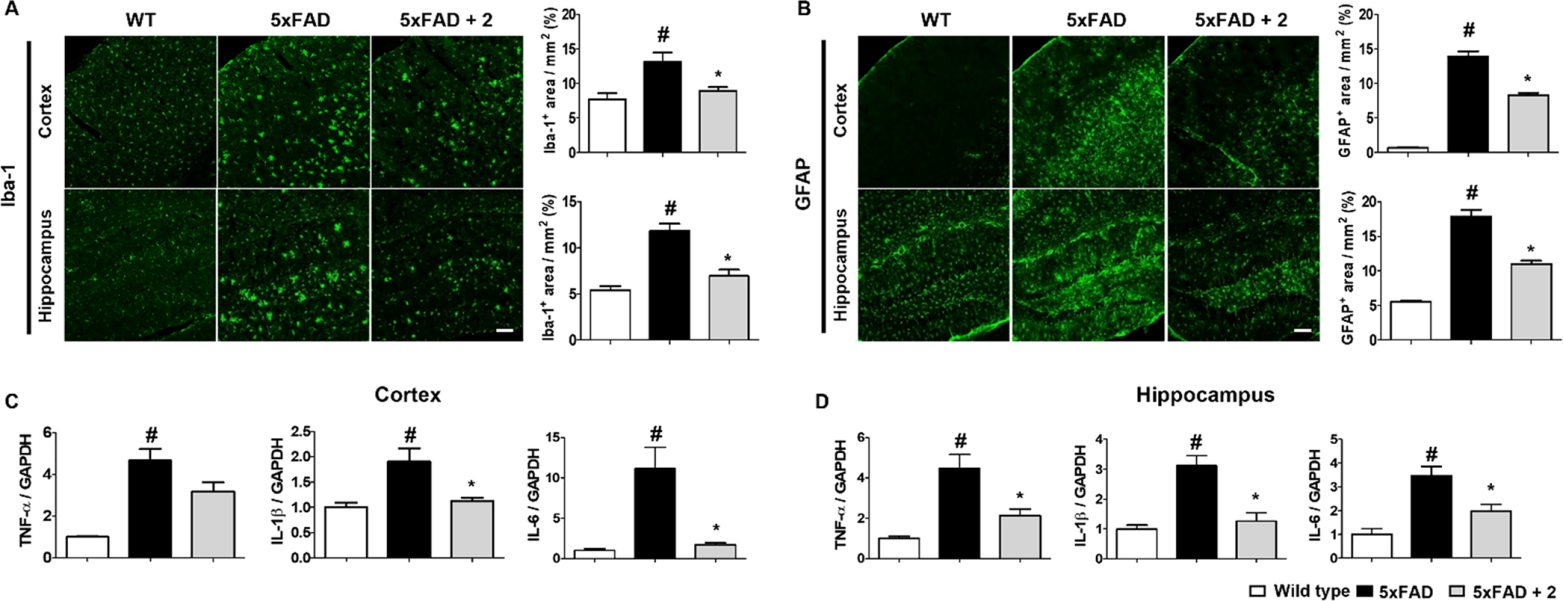
PRZ-18002 treatment diminished neuroinflammation and the production of proinflammatory cytokines. (A, B) Representative images and quantification data of Iba-1^+^ (A) and GFAP^+^ (B) staining in the brain. Microglia and astrocyte activations were elevated in vehicle-treated 5xFAD mice compared to wild-type mice, but these were decreased after PRZ-18002 treatment. Data are shown as mean ± SEM (Scale bar, 100 μm, WT; *n* = 4, 5xFAD; *n* = 5, 5xFAD+**2**; *n* = 5, one-way ANOVA, ^#^*P* < 0.05 vs wild-type, *P < 0.05 vs vehicle-treated 5xFAD). (C, D) mRNA levels of pro-inflammatory cytokine in mice brain. Expression of pro-inflammatory cytokines in 5xFAD group showed a significant increase compared with wild-type mice, but these were markedly decreased by PRZ-18002 treatment. Data are shown as mean ± SEM (WT; *n* = 4, 5xFAD; *n* = 5, 5xFAD+**2**; *n* = 5, one-way ANOVA, ^#^*P* < 0.05 vs wild-type, *P < 0.05 vs vehicle-treated 5xFAD).

We continued to investigate if PRZ-18002 could ameliorate AD pathologies in the mouse model, in which the 5xFAD mice aged 8 to 9 months old were treated with PRZ-18002 through intranasal injection under anesthetic conditions, which has been reported to exhibit minimal negative effects to the mice performance. We observed that PRZ-18002 was delivered to the brains of mice via intranasal injection, which remained up to 8 h (Figure S7). Treatment with PRZ-18002 for 1 month successfully reduced the level of p-p38 in the cortex and hippocampus of the 5xFAD mice (Figure 4A). In our previous study in the 9-months-old 5xFAD mice, we observed that a reduced level of p-p38 in the brain resulted in mitigated AD pathologies through regulation of neuroinflammatory conditions.^13^ Similarly, PRZ-18002 treatment significantly improved the Morris Water Maze (MWM) performance of the 9-month-old 5xFAD mice (Figure 4B–C), highlighting the reconstituting effect of PRZ-18002 on spatial memory and learning in the mice model. Moreover, we confirmed that PRZ-18002 effectively diminished the level of Aβ in the cortex and hippocampus of the 5xFAD mice (Figure 4D–E). Histologic analysis of Aβ using thioflavin-S and anti-amyloid beta antibody also showed that deposition of Aβ plaques was notably decreased in the 5xFAD mice treated with PRZ-18002 (Figure S8). Furthermore, neuroinflammation was shown to be markedly decreased in the 5xFAD mice treated with PRZ-18002 (Figure 5A–B). Consistent with previous observations in BV-2 cells (Figure 3F), we observed significant reduction in the mRNA level of proinflammatory cytokines, such as TNF-α, IL-1β and IL-6, in the cortex and hippocampus of the 5xFAD mice (Figure 5C–D). Collectively, these data suggest that PRZ-18002 alleviates neuroinflammation and pathophysiological hallmarks of AD, such as cognitive impairment and accumulation of Aβ, via inducing selective degradation of p-p38 and thereby downregulating proinflammatory signaling pathway.

### Decreased Phosphorylation of Tau by PRZ-18002 in the P301L/S320F Tau Expressing Cell and AD Mouse Model

**Figure 6:**
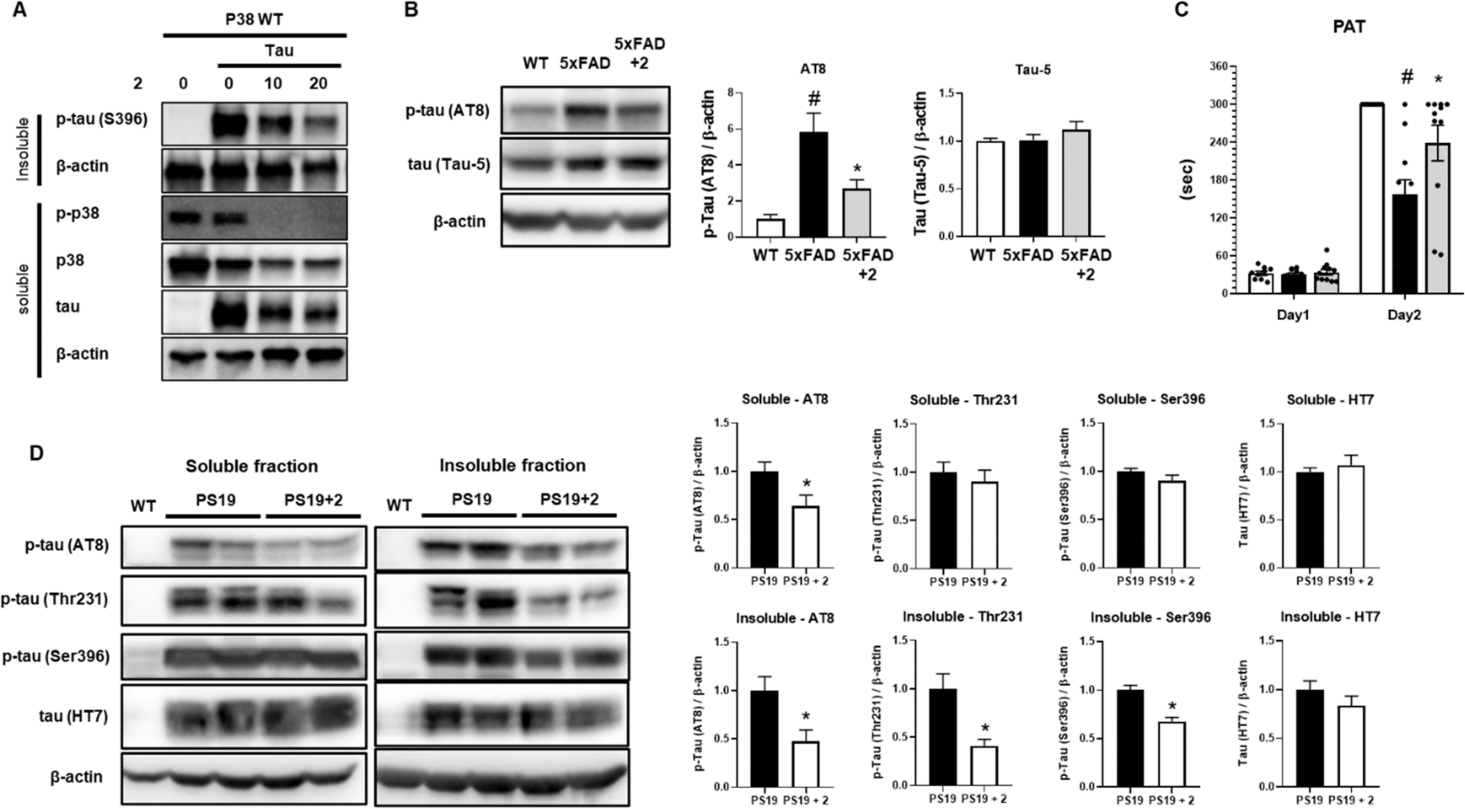
PRZ-18002 reduced aggregated p-tau protein in cells expressing tau P301L/S320F mutants and mice. (A) HEK293 cells were transfected with p38 wildtype plasmid together with or without tau P301L/S320F plasmid and incubated for 24 h. HEK293 cells overexpressing mutant tau were treated for 24 h with either compound [PRZ-18002 or 8b] or DMSO. Cell lysates were separated into an insoluble fraction and soluble fraction. Each sample of fractions was analyzed by immunoblotting using described antibodies. (B) Representative images and quantitative data of tau protein levels in the hippocampus of 5xFAD mice. Data are shown as mean ± SEM (*n* = 5 per group, one-way ANOVA, ^#^*P* < 0.05 vs wild-type, *P < 0.05 vs vehicle-treated 5xFAD) (C) Latency time record until entering to dark compartment in PAT. Maximum time was 300-s. (WT; *n* = 9, PS19; *n* = 11, PS19+**2**; *n* = 11, one-way ANOVA, ^#^*P* < 0.05 vs WT, *P < 0.05 vs PS19). (D) Representative images and quantitative data of tau protein levels in the hippocampus of PS19 mice. Data are shown as the mean ± SEM (*n* = 6 per group, *t* test, *P < 0.05 vs PS19).

Together with Aβ, hyper-phosphorylated tau (p-tau) is considered as one of the pathogenic proteins of AD. In order to confirm the efficacy of PRZ-18002 on tauopathy, we first conducted an experiment to determine whether the PRZ-18002 compound reduced the amount of p-tau at the cellular level. While P301L mutant tau is associated with frontotemporal dementia,^23^ S320F mutant tau is associated with Pick’s disease. It has been reported that tau phosphorylation increases when mutations occur in both sites of P301L and S320F.^24^ We thus used the cells ectopically expressing tau with P301L and S320F mutations and confirmed that PRZ-18002 reduced the level of p-tau protein (Figure 6A).

Given that p38 MAPK inhibition may contribute to reduction of p-tau level in a cellular model, we further investigated whether PRZ-18002 ameliorated tauopathy in a mouse model of AD. First, we measured the level of p-tau in 5xFAD mice. As expected, we found that the level of p-tau (AT8, Ser396) was elevated in 5xFAD mice compared to wild-type mice. We also found that treatment of PRZ-18002 decreased the level of p-tau, while that of total tau remained unchanged (Figure 6B). Next, when we performed the passive avoidance test (PAT) and measured the level of p-tau in a tau-based AD mouse model, PS19; 7-month-old mice entered the dark compartment at day 2, unaware of the foot shock of day 1. On the other hand, PS19 mice treated with PRZ-18002 remained at the bright compartment (Figure 6C). We also observed that PRZ-18002 significantly reduced the level of p-tau (AT8, Thr231, Ser396) in the insoluble fraction of the hippocampus of PS19 mice (Figure 6D). These results collectively demonstrate that PRZ-18002 may ameliorate tauopathy as well as Aβ pathology.

## Discussion

TPD has been recognized as an emerging therapeutic modality for innovative drug discovery in the recent decade. The drug-like properties of chemical degraders, such as PROTACs, have paved the way to deplete pathological proteins that have previously been challenging to target with conventional small molecule inhibitors or gene knockdown. In particular, pathologies associated with aberrant PTMs of disease-causing proteins can only be accessed by not genetic but protein knockdown. However, to date, a chemical degrader targeting PTMs of disease-relevant proteins, such as phosphorylation, has not been reported yet.

In this report, we have demonstrated that phosphorylated p38 can be targeted and sequentially eliminated by a TPD approach. For this study, we designed and synthesized small molecules preferentially targeting p-p38 over p38 by focusing on the glycine flip, a conformation that is readily formed in the hinge region of p-p38 and provides potent double hydrogen bonds with the ligands with the benzophenone moiety.^18,25^ Through the molecular modeling, we also demonstrated that PRZ-18002 could target the DFG-in motif, a characteristic of the fully phosphorylated kinase structure, providing selectivity to active p38. We used benzophenone derivatives as a protein-binding ligand and conjugated them through biologically compatible ethylene glycol or alkyl linkers to a CRBN ligand, pomalidomide, in order to hijack the E3 ubiquitin ligase complex. We identified PRZ-18002 as the most effective degrader of p-p38, which preferentially reduced p-p38 over its nonphosphorylated counterpart. The degradation was inhibited by proteasomal inhibitor or pomalidomide, indicating that the reduced protein level of p-p38 is due to CRBN-mediated ubiquitination and proteasomal degradation. While ectopic overexpression of kinase generally triggers activation of the kinase, we observed robust reduction in the level of the ectopically expressed phosphomimetic mutant of p38, highlighting selective and effective degradation of the activated form of p38 by PRZ-18002. To the best of our knowledge, we have presented, for the first time, a chemical degrader that directly targets a specific PTM of a disease-associated protein for proteasomal degradation, further advancing a TPD approach as a novel therapeutic strategy.

Previous studies using target protein degraders for neurodegenerative disease have focused on the direct removal of aggregated pathogenic proteins such as Aβ plaques, tau tangles, or mutant Huntingtins.^26^ Recently, regulating the inflammatory conditions in the brain has been suggested as a promising therapeutic strategy for AD.^27^ Moreover, the pivotal roles of p38 in the progression of AD have been extensively studied.^28−31^ Previous studies have demonstrated the increased level of p-p38 in conditions associated with AD, and p38 has been reported to be related to neuroinflammatory signals for aggravation of AD. In addition, other AD-related pathophysiologies have recently been disclosed to be associated with p38.^32−35^ For example, formation of tau aggregates is known to be accelerated by p38-mediated phosphorylation of tau. Also, it has been reported that Aβ can be scavenged via pharmacological inhibition of p38, collectively highlighting p38 as a therapeutic target of AD. A phase 2 study using a p38 inhibitor, Neflamapimod, showed that suppression of p38 can be a potential therapeutic strategy to treat AD.^36^ Although it has failed to accomplish the primary end point in clinical trials, treatment with Neflamapimod resulted in a significant decrease of p-tau and total tau in CSF and improvement of episodic memory function, as determined by the Hopkins verbal learning test.^12,37,38^ In particular, synaptic dysfunction was reported to be significantly improved. The p38 inhibitor has proven to be well tolerated in Phase I and II studies, conferring a novel strategy for AD treatment.^39−42^

Small molecules that target protein degradation against neurodegenerative disease remain an unconquered area. Small molecule protein degraders generally consist of a target binding warhead, a linker, and a E3 ligase binding ligand, thereby giving rise to a large molecular weight unsuitable as a CNS drug.^43^ Indeed, in our pilot trial via intraperitoneal injection, we observed neither significant improvement of memory function nor reduction of Aβ deposits and p-tau in AD mice treated with PRZ-18002, partly due to its large molecular weight hampering BBB penetration. Previously, several studies have shown that intranasal delivery of chemical compounds (MW > 500) can be an efficient approach to achieving brain delivery by bypassing the BBB.^44,45^ Furthermore, via intranasal administration combined with intravenous injection, tau-degrading peptide TH006 has shown to reduce the level of tau in the cortex of 3xTg mice.^46^ Interestingly, it has been reported that the efficiency of brain delivery via intranasal delivery is affected by head position, and it can be robustly improved by maintaining a “mecca position”.^47,48^ Therefore, we came up with an idea of intranasal administration of PRZ-18002 using a mouse positioning device, the Jerry seat, and successfully demonstrated that PRZ-18002 reduced the level of p-p38 in the brain of 9-month-old 5xFAD mice.^15^ Furthermore, PRZ-18002 diminished deposition of Aβ, hyper-phosphorylation of tau, reactive gliosis, and production of proinflammatory cytokines, thus resulting in improved spatial memory and learning in the AD mouse model.

Collectively, our findings suggest that TPD technology can target a specific PTM to induce selective degradation of neurodegenerative disease-associated proteins such as p-p38, demonstrating its potential as a therapeutic modality against AD.

## Supporting Information Available

Experimental details to prepare the chemicals and methods of biological assays; supplementary tables and figures (PDF)

## References

[ref1] Winter G. E.; Buckley D. L.; Paulk J.; Roberts J. M.; Souza A.; Dhe-Paganon S.; Bradner J. E. Phthalimide conjugation as a strategy for in vivo target protein degradation. Science 2015, 348, 1376–1381. 10.1126/science.aab1433. 25999370 PMC4937790

[ref2] Bondeson D. P.; Mares A.; Smith I. E. D.; Ko E.; Campos S.; Miah A. H.; Mulholland K. E.; Routly N.; Buckley D. L.; Gustafson J. L.; et al. Catalytic in vivo protein knockdown by small-molecule PROTACs. Nat. Chem. Biol. 2015, 11, 611–617. 10.1038/nchembio.1858. 26075522 PMC4629852

[ref3] Stanton B. Z.; Chory E. J.; Crabtree G. R. Chemically induced proximity in biology and medicine. Science 2018, 359, eaao5902 10.1126/science.aao5902. 29590011 PMC6417506

[ref4] Burslem G. M.; Crews C. M. Proteolysis-Targeting Chimeras as Therapeutics and Tools for Biological Discovery. Cell 2020, 181, 102–114. 10.1016/j.cell.2019.11.031. 31955850 PMC7319047

[ref5] Cattaneo A.; Chirichella M. Targeting the Post-translational Proteome with Intrabodies. Trends in Biotechnol. 2019, 37, 578–591. 10.1016/j.tibtech.2018.11.009. 30577991

[ref6] Pearson G.; Robinson F.; Beers Gibson T.; Xu B.-e.; Karandikar M.; Berman K.; Cobb M. H. Mitogen-Activated Protein (MAP) Kinase Pathways: Regulation and Physiological Functions. Endocr. Rev. 2001, 22, 153–183. 10.1210/edrv.22.2.0428. 11294822

[ref7] Johnson G. L.; Lapadat R. Mitogen-Activated Protein Kinase Pathways Mediated by ERK, JNK, and p38 Protein Kinases. Science 2002, 298, 1911–1912. 10.1126/science.1072682. 12471242

[ref8] Herlaar E.; Brown Z. p38 MAPK signalling cascades in inflammatory disease. Mol. Med. Today 1999, 5, 439–447. 10.1016/S1357-4310(99)01544-0. 10498912

[ref9] McLaughlin B.; Pal S.; Tran M. P.; Parsons A. A.; Barone F. C.; Erhardt J. A.; Aizenman E. p38 Activation Is Required Upstream of Potassium Current Enhancement and Caspase Cleavage in Thiol Oxidant-Induced Neuronal Apoptosis. J. Neurosci. 2001, 21, 3303–3311. 10.1523/JNEUROSCI.21-10-03303.2001. 11331359 PMC3746747

[ref10] Xie Y.; Tan Y.; Zheng Y.; Du X.; Liu Q. Ebselen ameliorates β-amyloid pathology, tau pathology, and cognitive impairment in triple-transgenic Alzheimer’s disease mice. JBIC, J. Biol. Inorg. Chem. 2017, 22, 851–865. 10.1007/s00775-017-1463-2. 28502066

[ref11] Zhao Y.-w.; Pan Y.-q.; Tang M.-m.; Lin W.-j. Blocking p38 Signaling Reduces the Activation of Pro-inflammatory Cytokines and the Phosphorylation of p38 in the Habenula and Reverses Depressive-Like Behaviors Induced by Neuroinflammation. Front. Pharmacol. 2018, 9, 511 10.3389/fphar.2018.00511. 29867510 PMC5962764

[ref12] Scheltens P.; Prins N.; Lammertsma A.; Yaqub M.; Gouw A.; Wink A. M.; Chu H.-M.; van Berckel B. N. M.; Alam J. An exploratory clinical study of p38α kinase inhibition in Alzheimer’s disease. Ann. Clin. Transl. Neurol. 2018, 5, 464–473. 10.1002/acn3.549. 29687023 PMC5899915

[ref13] Gee M. S.; Son S. H.; Jeon S. H.; Do J.; Kim N.; Ju Y.-J.; Lee S. J.; Chung E. K.; Inn K.-S.; Kim N.-J.; Lee J. K. A selective p38α/β MAPK inhibitor alleviates neuropathology and cognitive impairment, and modulates microglia function in 5XFAD mouse. Alzheimer’s Res. Ther. 2020, 12, 45 10.1186/s13195-020-00617-2. 32317025 PMC7175487

[ref14] Canovas B.; Nebreda A. R. Diversity and versatility of p38 kinase signalling in health and disease. Nat. Rev. Mol. Cell Biol. 2021, 22, 346–366. 10.1038/s41580-020-00322-w. 33504982 PMC7838852

[ref15] Ullah I.; Chung K.; Beloor J.; Lee S.-K.; Kumar P. A Positioning Device for the Placement of Mice During Intranasal siRNA Delivery to the Central Nervous System. JoVE 2019, e59201 10.3791/59201-v. 31475960

[ref16] Heo J.; Shin H.; Lee J.; Kim T.; Inn K.-S.; Kim N.-J. Synthesis and biological evaluation of N-cyclopropylbenzamide-benzophenone hybrids as novel and selective p38 mitogen activated protein kinase (MAPK) inhibitors. Bioorg. Med. Chem. Lett. 2015, 25, 3694–3698. 10.1016/j.bmcl.2015.06.036. 26115577

[ref17] Martz K. E.; Dorn A.; Baur B.; Schattel V.; Goettert M. I.; Mayer-Wrangowski S. C.; Rauh D.; Laufer S. A. Targeting the Hinge Glycine Flip and the Activation Loop: Novel Approach to Potent p38α Inhibitors. J. Med. Chem. 2012, 55, 7862–7874. 10.1021/jm300951u. 22897496

[ref18] Yurtsever Z.; Scheaffer S. M.; Romero A. G.; Holtzman M. J.; Brett T. J. The crystal structure of phosphorylated MAPK13 reveals common structural features and differences in p38 MAPK family activation. Acta Crystallogr., Sect. D 2015, 71, 790–799. 10.1107/S1399004715001212. 25849390 PMC4388263

[ref19] Hale K. K.; Trollinger D.; Rihanek M.; Manthey C. L. Differential Expression and Activation of p38 Mitogen-Activated Protein Kinase α, β, γ, and δ in Inflammatory Cell Lineages. J. Immunol. 1999, 162, 4246–4252. 10.4049/jimmunol.162.7.4246. 10201954

[ref20] Diskin R.; Askari N.; Capone R.; Engelberg D.; Livnah O. Active Mutants of the Human p38α Mitogen-activated Protein Kinase*. J. Biol. Chem. 2004, 279, 47040–47049. 10.1074/jbc.M404595200. 15284239

[ref21] Kirsch K.; Zeke A.; Toke O.; Sok P.; Sethi A.; Sebo A.; Kumar G. S.; Egri P.; Poti A. L.; Gooley P.; Peti W.; Bento I.; Alexa A.; Remenyi A. Co-regulation of the transcription controlling ATF2 phosphoswitch by JNK and p38. Nat. Commun. 2020, 11, 5769 10.1038/s41467-020-19582-3. 33188182 PMC7666158

[ref22] Petzold G.; Fischer E. S.; Thomä N. H. Structural basis of lenalidomide-induced CK1α degradation by the CRL4CRBN ubiquitin ligase. Nature 2016, 532, 127–130. 10.1038/nature16979. 26909574

[ref23] Hoover B. R.; Reed M. N.; Su J.; Penrod R. D.; Kotilinek L. A.; Grant M. K.; Pitstick R.; Carlson G. A.; Lanier L. M.; Yuan L.-L.; et al. Tau Mislocalization to Dendritic Spines Mediates Synaptic Dysfunction Independently of Neurodegeneration. Neuron 2010, 68, 1067–1081. 10.1016/j.neuron.2010.11.030. 21172610 PMC3026458

[ref24] Strang K. H.; Croft C. L.; Sorrentino Z. A.; Chakrabarty P.; Golde T. E.; Giasson B. I. Distinct differences in prion-like seeding and aggregation between Tau protein variants provide mechanistic insights into tauopathies. J. Biol. Chem. 2018, 293, 2408–2421. 10.1074/jbc.M117.815357. 29259137 PMC5818185

[ref25] Koeberle S. C.; Romir J.; Fischer S.; Koeberle A.; Schattel V.; Albrecht W.; Grütter C.; Werz O.; Rauh D.; Stehle T.; et al. Skepinone-L is a selective p38 mitogen-activated protein kinase inhibitor. Nat. Chem. Biol. 2012, 8, 141–143. 10.1038/nchembio.761. 22198732

[ref26] Wang Y.; Jiang X.; Feng F.; Liu W.; Sun H. Degradation of proteins by PROTACs and other strategies. Acta Pharm. Sin. B 2020, 10, 207–238. 10.1016/j.apsb.2019.08.001. 32082969 PMC7016280

[ref27] Kinney J. W.; Bemiller S. M.; Murtishaw A. S.; Leisgang A. M.; Salazar A. M.; Lamb B. T. Inflammation as a central mechanism in Alzheimer’s disease. Alzheimer Dement.: Transl. Res. Clin. Interv. 2018, 4, 575–590. 10.1016/j.trci.2018.06.014. PMC6214864 30406177

[ref28] Hensley K.; Floyd R. A.; Zheng N.-Y.; Nael R.; Robinson K. A.; Nguyen X.; Pye Q. N.; Stewart C. A.; Geddes J.; Markesbery W. R.; et al. p38 Kinase Is Activated in the Alzheimer’s Disease Brain. J. Neurochem. 1999, 72, 2053–2058. 10.1046/j.1471-4159.1999.0722053.x. 10217284

[ref29] Sun A.; Liu M.; Nguyen X. V.; Bing G. P38 MAP kinase is activated at early stages in Alzheimer’s disease brain. Exp. Neurol. 2003, 183, 394–405. 10.1016/S0014-4886(03)00180-8. 14552880

[ref30] Munoz L.; Ammit A. J. Targeting p38 MAPK pathway for the treatment of Alzheimer’s disease. Neuropharmacology 2010, 58, 561–568. 10.1016/j.neuropharm.2009.11.010. 19951717

[ref31] Lee J. K.; Kim N.-J. Recent Advances in the Inhibition of p38 MAPK as a Potential Strategy for the Treatment of Alzheimer’s Disease. Molecules 2017, 22, 1287 10.3390/molecules22081287. 28767069 PMC6152076

[ref32] Ittner A. A.; Gladbach A.; Bertz J.; Suh L. S.; Ittner L. M. p38 MAP kinase-mediated NMDA receptor-dependent suppression of hippocampal hypersynchronicity in a mouse model of Alzheimer’s disease. Acta Neuropathol. Commun. 2014, 2, 149 10.1186/s40478-014-0149-z. 25331068 PMC4212118

[ref33] Yu Q.; Du F.; Douglas J. T.; Yu H.; Yan S. S.; Yan S. F. Mitochondrial Dysfunction Triggers Synaptic Deficits via Activation of p38 MAP Kinase Signaling in Differentiated Alzheimer’s Disease Trans-Mitochondrial Cybrid Cells. J. Alzheimer’s Dis. 2017, 59, 223–239. 10.3233/JAD-170283. 28598851 PMC5935489

[ref34] Kheiri G.; Dolatshahi M.; Rahmani F.; Rezaei N. Role of p38/MAPKs in Alzheimer’s disease: implications for amyloid beta toxicity targeted therapy. Rev. Neurosci. 2018, 30, 9–30. 10.1515/revneuro-2018-0008. 29804103

[ref35] Muraleva N. A.; Stefanova N. A.; Kolosova N. G. SkQ1 Suppresses the p38 MAPK Signaling Pathway Involved in Alzheimer’s Disease-Like Pathology in OXYS Rats. Antioxidants 2020, 9, 676 10.3390/antiox9080676. 32731533 PMC7463502

[ref36] Prins N. D.; Harrison J. E.; Chu H.-M.; Blackburn K.; Alam J. J.; Scheltens P.; et al. A phase 2 double-blind placebo-controlled 24-week treatment clinical study of the p38 alpha kinase inhibitor neflamapimod in mild Alzheimer’s disease. Alzheimer’s Res. Ther. 2021, 13, 106 10.1186/s13195-021-00843-2. 34044875 PMC8157623

[ref37] Alam J.; Blackburn K.; Patrick D. Neflamapimod: Clinical Phase 2b-Ready Oral Small Molecule Inhibitor of p38α to Reverse Synaptic Dysfunction in Early Alzheimer′s Disease. J. Prev. Alzheimers Dis. 2017, 4, 273–278. 29181493 10.14283/jpad.2017.41

[ref38] Germann U. A.; Alam J. J. P38α MAPK Signaling—A Robust Therapeutic Target for Rab5-Mediated Neurodegenerative Disease. Int. J. Mol. Sci. 2020, 21, 5485 10.3390/ijms21155485. 32751991 PMC7432772

[ref39] Dong Y.; Li X.; Cheng J.; Hou L. Drug Development for Alzheimer’s Disease: Microglia Induced Neuroinflammation as a Target?. Int. J. Mol. Sci. 2019, 20, 558 10.3390/ijms20030558. 30696107 PMC6386861

[ref40] Fu W.-Y.; Wang X.; Ip N. Y. Targeting Neuroinflammation as a Therapeutic Strategy for Alzheimer′s Disease: Mechanisms, Drug Candidates, and New Opportunities. ACS Chem. Neurosci. 2019, 10, 872–879. 10.1021/acschemneuro.8b00402. 30221933

[ref41] Lee G.; Cummings J.; Decourt B.; Leverenz J. B.; Sabbagh M. N. Clinical drug development for dementia with Lewy bodies: past and present. Exp. Opin. Invest. Drugs 2019, 28, 951–965. 10.1080/13543784.2019.1681398. PMC6823159 31614096

[ref42] Cummings J.; Lee G.; Ritter A.; Sabbagh M.; Zhong K. Alzheimer’s disease drug development pipeline: 2020. Alzheimer′s Dement.: Transl. Res. Clin. Interv. 2020, 6, e12050 10.1002/trc2.12050. PMC7364858 32695874

[ref43] Ding Y.; Fei Y.; Lu B. Emerging New Concepts of Degrader Technologies. Trends Pharmacol. Sci. 2020, 41, 464–474. 10.1016/j.tips.2020.04.005. 32416934 PMC7177145

[ref44] Dhuria S. V.; Hanson L. R.; Frey W. H. 2nd Intranasal delivery to the central nervous system: mechanisms and experimental considerations. J. Pharm. Sci. 2010, 99, 1654–1673. 10.1002/jps.21924. 19877171

[ref45] Ullah I.; Chung K.; Oh J.; Beloor J.; Bae S.; Lee S. C.; Lee M.; Kumar P.; Lee S. K. Intranasal delivery of a Fas-blocking peptide attenuates Fas-mediated apoptosis in brain ischemia. Sci. Rep. 2018, 8, 15041 10.1038/s41598-018-33296-z. 30301943 PMC6178348

[ref46] Chu T. T.; Gao N.; Li Q. Q.; Chen P. G.; Yang X. F.; Chen Y. X.; Zhao Y. F.; Li Y. M. Specific Knockdown of Endogenous Tau Protein by Peptide-Directed Ubiquitin-Proteasome Degradation. Cell Chem. Biol. 2016, 23, 453–461. 10.1016/j.chembiol.2016.02.016. 27105281

[ref47] Merkus P.; Ebbens F. A.; Muller B.; Fokkens W. J. Influence of anatomy and head position on intranasal drug deposition. Eur. Arch. Oto-Rhino-Laryngol. 2006, 263, 827–832. 10.1007/s00405-006-0071-5. 16807754

[ref48] Wu H.; Hu K.; Jiang X. From nose to brain: understanding transport capacity and transport rate of drugs. Exp. Opin. Drug Delivery 2008, 5, 1159–1168. 10.1517/17425247.5.10.1159. 18817519

