# Supplementary material for: Chemical Knockdown of Phosphorylated p38 Mitogen-Activated Protein Kinase (MAPK) as a Novel Approach for the Treatment of Alzheimer′s Disease
Source: ACS Cent Sci. 2023 Mar 1;9(3):417–26. doi: 10.1021/acscentsci.2c01369 (PMC10037464; doi:10.1021/acscentsci.2c01369)
Supplement: Supplementary file 1 — oc2c01369_si_001.pdf [file oc2c01369_si_001.pdf]

Supporting Information

Chemical Knockdown of Phosphorylated p38 Mitogen-Activated Protein Kinase (MAPK) as a Novel Approach for the Treatment of Alzheimer's Disease

Seung Hwan Son,[a,‡] Na-Rae Lee,[b,‡] Min Sung Gee,[a,‡] Chae Won Song,[b] Soo Jin Lee,[a] Sang-Kyung Lee,[c] Yoonji Lee,[d] Hee Jin Kim,[b] Jong Kil Lee,[a,b,*] Kyung-Soo Inn,[a,b,*] and Nam-Jung Kim[a,b,*]

[a]College of Pharmacy, Kyung Hee University, Seoul 02447, Republic of Korea

[b]Prazer Therapeutics Inc., Beobwon-ro 9-gil 26, Songpa-gu, Seoul 05836, Republic of Korea

[c]Department of Bioengineering and Institute of Nanoscience and Technology, Hanyang University, Seoul 04763, Republic of Korea

[d]College of Pharmacy, Chung-Ang University, Seoul, 06974, Republic of Korea

*E mail: jklee3984@khu.ac.kr (J.K.L).
*E mail: innks@khu.ac.kr (K.-S.I).
*E mail: kimnj@khu.ac.kr (N.-J.K).

[‡]These authors contributed equally

Table of Contents

1. **General Information** S3
2. **Chemical** S4
   2.1. **Synthesis of p-p38 MAPK degraders 1-7** S4
   2.2. **Synthesis of Compound 14 and 16** S11
3. **Biological Assay** S12
4. **Molecular Modeling Studies** S15
5. **Supplementary Tables** S15
   5.1. **Table S1. Information of antibodies used in this study** S15
   5.2. **Table S2. Information of qRT-PCR primers used in this study** S17
6. **Supplementary Figures** S18
7. **References** S24

1. General Information

Unless stated otherwise, all solvents and commercial reagents were purchased from commercial suppliers (Sigma Aldrich, TCI, Alfa Aesar and Acros Organics) and used without further purification. All reactions were performed under argon atmosphere. ^{1}H NMR was measured and obtained using a Bruker 400 and ^{13}C{^{1}H}-NMR spectra were measured on a Varian VNMRS500 spectrometer. ^{1}H and ^{13}C{^{1}H}-NMR chemical shifts were determined relative to the signal of the residual solvent peak used as an internal reference. Signals are recorded as follows: chemical shift (δ, ppm), multiplicity (s = singlet, d = doublet, t = triplet, m = multiplet, br = broad). Coupling constants (J) were reported in Hertz (Hz). High-resolution mass spectrometry (HRMS) data were recorded using a JEOL JMS-700 instrument in the EI or ESI mode and Jeol AccuTOF (JMS-T100TD) equipped with a DART (direct analysis in real time) ion source from Ionsens, (Tokyo, Japan) in the positive modes. Liquid Chromatography-Mass Spectrometry (LCMS) data were recorded on an Agilent 6130 + HP1100. Flash column chromatography was performed using silica gel 60 (230–400mesh) and analytical thin layer chromatography (TLC) was performed using TLC Silica gel 60 F254.

2. Chemical

2.1. Synthesis of p-p38 MAPK degraders 1-7

The compound **11** and **13** was prepared according to the literature previously reported.[1]

(4-Bromophenyl)(4-(2-hydroxyethoxy)phenyl)methanone (12a)[1]

To a solution of (4-bromophenyl)(4-hydroxyphenyl)methanone (**11)** (350 mg, 1.270 mmol, 1 eq) in acetone was added K_2CO_3 (2.5 eq) and 2-bromoethanol (2.75 eq). The reaction mixture was refluxed for overnight, quenched with 2 N HCl and diluted with EtOAc. The combined organic layer was washed with H_2O, dried over $MgSO_4$, and concentrated in vacuo. The residue was purified by column chromatography on silica (EtOAc/*n*-hexane = 1/5 to 1/2). Compound **12a** (260 mg, 64%) was obtained as a white solid. ^{1}H-NMR (400 MHz, $CDCl_3$) δ 7.80 (d, *J* = 8.8 Hz, 2H), 7.60 – 7.65 (m, 4H), 6.99 (d, *J* = 8.8 Hz, 2H), 4.19 – 4.17 (m, 2H), 4.03 – 4.01 (m, 2H); HR-MS (ESI$^+$) calculated for [M$^+$] 320.0043; found 320.0020.

(4-Bromophenyl)(4-(2-(2-hydroxyethoxy)ethoxy)phenyl)methanone (12b)[1]

Compound **12b** (424 mg, 86%) was obtained as a white solid from (4-bromophenyl)(4-hydroxyphenyl)methanone (**11**) (376 mg, 1.35 mmol) using the procedure similar to that for **12a**, except that 2-(2-bromoethoxy)ethanol was used. ^{1}H-NMR (400 MHz, $CDCl_3$) δ 7.81 – 7.78 (m, 4H), 7.62 – 7.47 (m, 2H), 7.00 – 6.98 (m, 2H), 4.24 – 4.22 (m, 2H), 3.93 – 3.90 (m, 2H), 3.80 – 3.78 (m, 2H), 3.71 – 3.69 (m, 2H); HR-MS (ESI$^+$) calculated for [M$^+$] 364.0305; found 364.0296.

(4-Bromophenyl)(4-((5-hydroxypentyl)oxy)phenyl)methanone (12c)

Compound **12c** (72 mg, 39%) was obtained as a solid from (4-bromophenyl)(4-hydroxyphenyl)methanone (**11**) (141 mg, 0.511 mmol) using the procedure similar to that for **12a**, except that 5-bromopentan-1-ol was used. ^{1}H-NMR (400 MHz, $CDCl_3$) δ 7.75 (dd, *J* = 7.1,5.7 Hz, 2H), 7.59 (d, *J* = 5.8 Hz, 4H), 6.92 (t, *J* = 7.4 Hz, 2H), 4.02 (d, *J* = 6 Hz, 2H), 3.65 (t, *J* = 6.1 Hz, 2H), 2.90 (m, dd, *J* = 31.7, 5.8 Hz, 2H), 1.86 (d, *J* = 5.9 Hz, 2H), 1.62 (t, *J* = 6.2 Hz, 2H).

(4-Bromophenyl)(4-(2-(2-(2-hydroxyethoxy)ethoxy)ethoxy)phenyl)methanone (12d)

Compound **12d** (235 mg, 98%) was obtained as a solid from (4-bromophenyl)(4-hydroxyphenyl)methanone (**11**) (150 mg, 0.541 mmol) using the procedure similar to that for **12a**, except that 2-(2-(2-bromoethoxy)ethoxy)ethanol was used. ^{1}H-NMR (400 MHz, $CDCl_3$) δ 7.78 (d, *J* = 8.6 Hz, 2H), 7.62 (s, 4H), 6.98 (d, *J* = 8.6 Hz, 2H), 4.22 (t, *J* = 4.6 Hz, 2H), 3.90 (t, *J* = 4.5 Hz, 2H), 3.77-3.68 (m, 6H), 3.62 (t, *J* = 4.3 Hz, 2H).

N-Cyclopropyl-4′-(4-(2-hydroxyethoxy)benzoyl)-6-methyl-[1,1′-biphenyl]-3-carboxamide (8a)[1]

To a solution of **12a** (223 mg, 0.696 mmol, 1 eq) and **13** (1 eq) in DMF (2 mL) was added K_2CO_3 (2 eq) and $Pd(PPh_3)_4$ (0.08 eq) at ambient temperature. After stirring for overnight at 90 °C, the reaction mixture was cooled to rt, quenched with 2 N HCl, and diluted with EtOAc. The combined organic layer was washed with brine, H_2O, dried over $MgSO_4$, and concentrated in vacuo. The residue was purified by column chromatography on silica (EtOAc/*n*-hexane = 1/2 to 2/1). Compound **8a** (190 mg, 66%) was obtained as a white solid. ^{1}H-NMR (400 MHz, $CDCl_3$) δ 7.88 (d, J = 8.6 Hz, 1H), 7.82 (d, J = 8.0 Hz, 1H),7.68 – 7.63 (m, 2H), 7.55 (d, J = 7.4 Hz, 1H), 7.44 (d, J = 8.0 Hz, 3H), 7.35 (d, J = 8.0 Hz, 1H), 7.02 (d, J = 8.5 Hz, 2H), 4.20 (t, J = 4.3 Hz, 2H), 4.03 (t, J = 4.3 Hz, 2H), 2.92 (m, 1H), 2.33 (s, 3H), 0.88 – 0.83 (m, 2H), 0.63 – 0.62 (m, 2H); HR-MS (ESI$^+$) calculated for [M$^+$] 415.1778; found 415.1793.

N-Cyclopropyl-4′-(4-(2-(2-hydroxyethoxy)ethoxy)benzoyl)-6-methyl-[1,1′-biphenyl]-3-carboxamide (8b)[1]

Compound **8b** (181 mg, 75%) was obtained as a white solid from **12b** (394 mg, 1.08 mmol) using the procedure similar to that for **8a**. ^{1}H-NMR (400 MHz, $CDCl_3$) δ 7.84 (d, J = 8.6, Hz, 2H), 7.80 (d, J = 8.3 Hz, 1H), 7.69 – 7.64 (m, 3H), 7.39 (d, J = 8.0 Hz, 2H), 7.32 (d, J = 7.8 Hz, 1H), 7.00 (d, J = 8.6 Hz, 2H), 6.55 (s, 1H), 4.23 (d, J = 3.6 Hz, 2H), 3.91 (d, J = 3.6 Hz, 2H), 3.80 (t, J = 4.3 Hz, 2H), 3.70 (t, J = 4.3 Hz, 2H), 2.90 (m, 1H), 2.31 (s, 3H), 0.85 (d, J = 6.4 Hz, 2H), 0.63 (s, 2H); HR-MS (ESI$^+$) calculated for [M+H$^+$] 460.2119; found 460.2152.

N-Cyclopropyl-4′-(4-((5-hydroxypentyl)oxy)benzoyl)-6-methyl-[1,1′-biphenyl]-3-carboxamide (8c)

Compound **8c** (47 mg, 60%) was obtained as a white solid from **12c** (63 mg, 0.174 mmol) using the procedure similar to that for **8a**. ^{1}H-NMR (400 MHz, $CDCl_3$) δ 7.80 (dd, J = 21.1, 8.3 Hz, 3H), 7.70-7.60 (m, 3H), 7.53 (m, 1H), 7.44 (td, J = 7.4,2.5 Hz, 1H), 7.38 (d, J = 8 Hz, 1H), 7.30 (d, J = 7.9 Hz, 1H), 6.95 (d, J = 8.6 Hz, 1H), 6.65 (s, 1H), 4.04 (t, J = 6.3 Hz, 2H), 3.67 (t, J = 6.3 Hz, 2H), 2.88 (m, 1H), 2.29 (s, 3H), 1.89-1.79 (m, 3H), 1.69-1.61 (m, 3H), 1.60-1.50 (m, 3H), 0.82 (d, J = 5.7 Hz, 2H), 0.63-0.58 (m, 2H).

N-Cyclopropyl-4′-(4-(2-(2-(2-hydroxyethoxy)ethoxy)ethoxy)benzoyl)-6-methyl-[1,1′-biphenyl]-3-carboxamide (8d)

Compound **8d** (100 mg, 35%) was obtained as a white solid from **12d** (235 mg, 0.573 mmol) using the procedure similar to that for **8a**. ^{1}H-NMR (400 MHz, $CDCl_3$) δ 7.11 (dd, J = 8.7 Hz, 4H), 7.67 (d, J = 7.8 Hz, 1H), 7.62 (s, 1H), 7.38 (d, J = 7.9 Hz, 2H), 7.31 (d, J = 7.8 Hz, 1H), 6.99 (d, J = 8.6 Hz, 2H), 6.54 (s, 1H), 4.21 (t, J = 4.4 Hz, 2H), 3.90 (t, J = 4.3 Hz, 2H), 3.71 (t, J = 6.1 Hz, 6H), 3.61 (t, J = 4.4 Hz, 2H), 2.87 (m, 1H), 2.30 (s, 3H), 0.84 (m, 2H), 0.57 (m, 2H).

2-(4-(5′-(Cyclopropylcarbamoyl)-2′-methyl-[1,1′-biphenyl]-4-carbonyl)phenoxy)ethyl (4-nitrophenyl) carbonate (9a)

To a solution of 4-nitrophenyl carbonochloridate (5 eq) in dry CH_2Cl_2 (1 mL) was added compound **8a** (93 mg, 0.224 mmol, 1 eq) and dry pyridine (5 eq) in dry CH_2Cl_2 (1 mL) at 0 °C. The reaction mixture was stirred until compound **8a** disappeared. Then the mixture was quenched with 2 N HCl and diluted with EtOAc. The combined organic layer was washed with H_2O, dried over $MgSO_4$, and concentrated in vacuo. The residue was purified by column chromatography on silica (EtOAc/*n*-hexane = 1/3 to 2/1). Compound **9a** (105 mg, 81%) was obtained as a white solid. ^{1}H-NMR (400 MHz, $CDCl_3$) δ 8.27 (d, J = 9.2 Hz, 2H), 7.86 (d, J = 8.8 Hz, 2H), 7.78 (d, J = 8.4 Hz, 2H), 7.70 – 7.62 (m, 2H), 7.39 (d, J = 8.9 Hz, 4H), 7.31 (d, J = 7.8 Hz, 1H), 7.02 (d, J = 8.8 Hz, 2H), 6.58 (s, 1H), 4.68 (d, J = 5.3 Hz, 2H), 4.37 (d, J = 5.2 Hz, 2H), 2.92 – 2.84 (m, 1H), 2.30 (s, 3H), 0.83 (d, J = 7.0 Hz, 2H), 0.64 – 0.57 (m, 2H).

2-(2-(4-(5′-(Cyclopropylcarbamoyl)-2′-methyl-[1,1′-biphenyl]-4-carbonyl)phenoxy)ethoxy)ethyl (4-nitrophenyl) carbonate (9b)

Compound **9b** (123 mg, 89%) was obtained as a white solid from **8b** (101 mg, 0.220 mmol) using the procedure similar to that for **9a**. ^{1}H-NMR (400 MHz, $CDCl_3$) δ 8.26 (d, J = 9.0 Hz, 2H), 7.84 (dd, J = 20.6, 8.2 Hz, 4H), 7.67 (d, J = 7.7 Hz, 1H), 7.62 (s, 1H), 7.42 (d, J = 7.8 Hz, 2H), 7.36 (t, J = 8.8 Hz, 3H), 7.02 (d, J = 8.6 Hz, 2H), 6.27 (s, 1H), 4.48 (d, J = 4.2 Hz, 2H), 4.26 (d, J = 4.0 Hz, 2H), 3.93 (dd, J = 17.8, 3.9 Hz, 4H), 2.90 (m, 1H), 2.32 (s, 3H), 0.87 (d, J = 6.4 Hz, 2H), 0.62 (s, 2H).

2-(2-(2-(4-(5′-(Cyclopropylcarbamoyl)-2′-methyl-[1,1′-biphenyl]-4-carbonyl)phenoxy)ethoxy)ethoxy)ethyl (4-nitrophenyl) carbonate (9d)

Compound **9d** (132 mg, 66%) was obtained as a white solid from **8d** (150 mg, 0.299 mmol) using the procedure similar to that for **9a**. ^{1}H-NMR (400 MHz, $CDCl_3$) δ 8.24 (d, J = 9.1 Hz, 2H), 7.81 (dd, J = 20.4, 8.4 Hz, 4H), 7.67 (dd, J = 7.8, 1.6 Hz, 1H), 7.63 (d, J = 1.4 Hz, 1H), 7.42 – 7.30 (m, 4H), 6.98 (d, J = 8.8 Hz, 1H), 6.49 (s, 1H), 4.46 – 4.41 (m, 2H), 4.22 (t, J = 4.6 Hz, 2H), 3.94 – 3.87 (m, 2H), 3.84 – 3.79 (m, 2H), 3.79 – 3.70 (m, 4H), 2.89 (m, 1H), 0.84 (dd, J = 12.6, 6.8 Hz, 2H), 0.63-0.58 (m, 2H).

2-(4-(5′-(Cyclopropylcarbamoyl)-2′-methyl-[1,1′-biphenyl]-4-carbonyl)phenoxy)ethyl 4-methylbenzenesulfonate (10a)

To a solution of compound **8a** (50 mg, 0.128 mmol, 1 eq) and triethylamine (3.5 eq) in CH_2Cl_2 was added *p*-toluenesulfonyl chloride (1.2 eq) at 0 °C. Then the reaction mixture was stirred at room temperature until compound **8a** disappeared. The mixture was quenched with water and diluted with EtOAc. The combined organic layer was washed with H_2O, dried over $MgSO_4$, and concentrated in vacuo. The residue was purified by column chromatography on silica (EtOAc/*n*-hexane = 1/1 to 2/1). Compound **10a** (47 mg, 65%) was obtained as a white solid. ^{1}H-NMR (400 MHz, $CDCl_3$) δ 7.91 – 7.78 (m, 6H), 7.67 (dd, J = 7.9, 1.3 Hz, 1H), 7.63 (d, J =

1.4 Hz, 1H), 7.42 (d, *J* = 8.1 Hz, 2H), 7.35 (t, *J* = 7.1 Hz, 3H), 7.01 (d, *J* = 8.8 Hz, 1H), 6.88 (d, *J* = 8.8 Hz, 2H), 6.29 (s, 1H), 4.51 – 4.37 (m, 2H), 4.30 – 4.22 (m, 2H), 2.91 (m, 1H), 2.46 (s, 3H), 2.32 (s, 3H), 0.87 (d, *J* = 6.3 Hz, 2H), 0.65 – 0.58 (m, 2H).

2-(2-(4-(5′-(Cyclopropylcarbamoyl)-2′-methyl-[1,1′-biphenyl]-4-carbonyl)phenoxy)ethoxy)ethyl 4-methylbenzenesulfonate (10b)

Compound **10b** (38 mg, 61%) was obtained as a white solid from **8b** (47 mg, 0.102 mmol) using the procedure similar to that for **10a**. ^{1}H-NMR (400 MHz, $CDCl_3$) *δ* 7.89 – 7.75 (m, 6H), 7.67 (d, *J* = 8.0 Hz, 1H), 7.62 (s, 1H), 7.42 (d, *J* = 7.8 Hz, 2H), 7.38 – 7.29 (m, 3H), 6.99 (d, *J* = 8.6 Hz, 2H), 6.27 (s, 1H), 4.17 (dd, *J* = 19.4, 4.1 Hz, 4H), 3.87-3.75 (m, 4H), 2.90 (m, 1H), 2.42 (s, 3H), 2.32 (s, 3H), 0.87 (d, *J* = 6.2 Hz, 2H), 0.62 (s, 2H).

5-(4-(5′-(Cyclopropylcarbamoyl)-2′-methyl-[1,1′-biphenyl]-4-carbonyl)phenoxy)pentyl 4-methylbenzenesulfonate (10c)

Compound **10c** (38 mg, 61%) was obtained as a white solid from **8c** (47 mg, 0.102 mmol) using the procedure similar to that for **10a**. ^{1}H-NMR (400 MHz, $CDCl_3$) *δ* 7.83 (d, *J* = 8.7 Hz, 2H), 7.78 (q, *J* = 3.8 Hz, 4H), 7.67 (dd, *J* = 7.9, 1.6 Hz, 1H), 7.63 (d, *J* = 1.5 Hz, 1H), 7.39 (d, *J* = 8.1 Hz, 2H), 7.32 (t, *J* = 7.0 Hz, 3H), 6.93 (d, *J* = 8.7 Hz, 2H), 6.49 (s, 1H), 4.06 (t, *J* = 6.3 Hz, 2H), 4.00 (t, *J* = 6.2 Hz, 2H), 2.88 (m, 1H), 2.43 (s, 3H), 2.30 (s, 3H), 1.81 – 1.67 (m, 4H), 1.57 – 1.46 (m, 2H), 0.87 – 0.80 (m, 2H), 0.64 – 0.58 (m, 2H).

2-(2-(2-(4-(5′-(Cyclopropylcarbamoyl)-2′-methyl-[1,1′-biphenyl]-4-carbonyl)phenoxy)ethoxy)ethoxy)ethyl 4-methylbenzenesulfonate (10d)

Compound **10d** (48 mg, 74%) was obtained as a white solid from **8d** (50 mg, 0.099 mmol) using the procedure similar to that for **10a**. ^{1}H-NMR (400 MHz, $CDCl_3$) *δ* 7.83 (d, *J* = 8.6 Hz, 2H), 7.77 (dd, *J* = 8.1, 2.1 Hz, 4H), 7.68 (d, *J* = 7.9 Hz, 1H), 7.63 (s, 1H), 7.38 (d, *J* = 7.9 Hz, 2H), 7.31 (d, *J* = 7.9 Hz, 3H), 6.98 (d, *J* = 8.6 Hz, 2H), 6.58 (s, 1H), 4.19 (t, *J* = 4.3 Hz, 2H), 4.14 (t, *J* = 4.7 Hz, 2H), 3.85 (t, *J* = 4.3 Hz, 2H), 3.71 – 3.64 (m, 4H), 3.63 – 3.59 (m, 2H), 2.87 (m, 1H), 2.41 (s, 3H), 2.29 (s, 3H), 0.82 (d, *J* = 6.0 Hz, 2H), 0.63 – 0.57 (m, 2H).

2-(4-(5′-(Cyclopropylcarbamoyl)-2′-methyl-[1,1′-biphenyl]-4-carbonyl)phenoxy)ethyl (2-(2,6-dioxopiperidin-3-yl)-1,3-dioxoisoindolin-4-yl)carbamate (1)

To a solution of compound **14a** (50 mg, 0.086 mmol, 1 eq) and pomalidomide **9** (1 eq) in dry DMF (1 mL) was added NaH (1.5 eq) slowly at 0 °C under the argon atmosphere. The reaction mixture was stirred at room temperature until compound **14a** disappeared. The mixture was quenched with NH_4Cl aqueous solution at 0 °C and diluted with EtOAc. The combined organic layer was washed with H_2O, dried over $MgSO_4$, and concentrated in vacuo. The residue was purified by column chromatography on silica (EtOAc/*n*-hexane = 1/1 to 4/1). Compound **1** (24 mg, 40%) was obtained as a white solid. ^{1}H-NMR (400 MHz, DMSO-d_6) *δ* 11.16 (s, 1H), 9.22

(s, 1H), 8.46 (s, 1H), 8.24 (d, J = 8.3 Hz, 1H), 7.86 (t, J = 7.8 Hz, 1H), 7.78 (q, J = 7.9 Hz, 5H), 7.60 (d, J = 7.2 Hz, 1H), 7.57 (d, J = 7.9 Hz, 2H), 7.42 (d, J = 7.8 Hz, 1H), 7.18 (d, J = 8.6 Hz, 2H), 5.13 (dd, J = 12.5, 5.2 Hz, 1H), 4.54 (s, 1H), 4.40 (s, 1H), 2.86 (m, 2H), 2.58 (d, J = 17.8 Hz, 2H), 2.31 (s, 3H), 2.04 (m, 1H), 1.99 (s, 1H), 0.68 (d, J = 6.7 Hz, 2H), 0.56 (s, 2H); $^{13}C\{^1H\}$-NMR (500 MHz, $CDCl_3$) δ 195.3, 170.8, 168.9, 168.6, 167.9, 162.1, 152.8, 145.0, 141.2, 139.3, 137.8, 137.0, 136.6, 132.7(2C), 132.2, 131.5, 130.9, 130.8, 130.0(2C), 129.2(2C), 128.2, 126.3, 123.9, 118.1, 115.1, 114.4(2C), 66.2, 64.1, 49.4, 31.5, 23.3, 22.7, 20.6, 6.9(2C); LR-MS (ESI$^+$) calculated for [M+H$^+$] 715.2; found 715.4.

2-(2-(4-(5′-(Cyclopropylcarbamoyl)-2′-methyl-[1,1′-biphenyl]-4-carbonyl)phenoxy)ethoxy)ethyl (2-(2,6-dioxopiperidin-3-yl)-1,3-dioxoisoindolin-4-yl)carbamate (2)

Compound **2** (26 mg, 30%) was obtained as a white solid from **14b** (70 mg, 0.112 mmol) using the procedure similar to that for **1**. ^1H-NMR (400 MHz, DMSO-d_6) δ 11.12 (s, 1H), 9.04 (s, 1H), 8.41 (s, 1H), 8.20 (d, J = 8.3 Hz, 1H), 7.71 (d, J = 10.3 Hz, 6H), 7.51 (d, J = 7.8 Hz, 3H), 7.37 (d, J = 7.7 Hz, 1H), 7.06 (d, J = 8.5 Hz, 2H), 5.08 (m, 1H), 4.22 (d, J = 33.8 Hz, 4H), 3.76 (d, J = 27 Hz, 4H), 2.86 – 2.74 (m, 2H), 2.54 (d, J = 19.5 Hz, 1H), 2.26 (s, 3H), 2.03-1.91 (m, 2H), 0.63 (d, J = 6.9 Hz, 2H), 0.51 (d, J = 1.3 Hz, 2H); $^{13}C\{^1H\}$-NMR (500 MHz, $CDCl_3$) δ 195.2, 170.9, 168.8, 168.6, 168.0, 166.7, 162.6, 153.1, 144.9, 141.2, 138.3, 137.9, 137.1, 136.5, 132.7(2C), 132.3, 131.4, 130.9, 130.3(2C), 129.9(2C), 129.1, 128.2, 126.3, 123.8, 118.0, 115.1, 114.4(2C), 69.8(2C), 67.5, 65.0, 49.3, 31.5, 23.5, 22.3, 20.8, 6.6(2C); LR-MS (ESI$^+$) calculated for [M+H$^+$] 759.3; found 759.3.

2-(2-(2-(4-(5′-(Cyclopropylcarbamoyl)-2′-methyl-[1,1′-biphenyl]-4-carbonyl)phenoxy)ethoxy)ethoxy)ethyl (2-(2,6-dioxopiperidin-3-yl)-1,3-dioxoisoindolin-4-yl)carbamate (3)

Compound **3** (49 mg, 55%) was obtained as a white solid from **14d** (74 mg, 0.111 mmol) using the procedure similar to that for **1**. ^1H-NMR (400 MHz, DMSO-d_6) δ 11.12 (s, 1H), 9.04 (s, 1H), 8.40 (s, 1H), 8.20 (d, J = 8.3 Hz, 1H), 7.80 – 7.64 (m, 6H), 7.51 (d, J = 7.2 Hz, 3H), 7.37 (d, J = 7.5 Hz, 1H), 7.04 (d, J = 7.5 Hz, 2H), 5.07 (m, 1H), 4.18 (d, J = 32.2 Hz, 4H), 3.69 (d, J = 41 Hz, 4H), 3.57 (s, 4H), 2.88 – 2.73 (m, 2H), 2.53 (d, J = 19.4 Hz, 1H), 2.26 (s, 3H), 2.07 – 1.87(m, 2H), 0.63 (d, J = 6.3 Hz, 2H), 0.50 (s, 2H); $^{13}C\{^1H\}$-NMR (500 MHz, $CDCl_3$) δ 195.3, 170.8, 168.8, 168.6, 168.0, 166.8, 162.6, 153.0, 141.2, 137.9, 137.0, 136.5, 132.6(2C), 132.2, 130.8(2C), 129.9(2C), 129.1, 128.2, 126.4, 123.8, 117.9, 115.0, 114.3(2C), 70.6, 69.7, 69.2, 67.7, 65.1, 60.5, 49.3, 31.5, 23.3, 22.7, 20.6, 6.8(2C); LR-MS (ESI$^+$) calculated for [M+H$^+$] 803.3; found 803.3.

***N*-Cyclopropyl-4′-(4-(2-((2-(2,6-dioxopiperidin-3-yl)-1,3-dioxoisoindolin-4-yl)oxy)ethoxy)benzoyl)-6-methyl-[1,1′-biphenyl]-3-carboxamide (4)**

To a solution of compound **15a** (63 mg, 0.111 mmol, 1 eq) and compound **10** (0.8 eq) in MeCN

(1 mL) was added K_2CO_3 (1.5 eq). The reaction mixture was refluxed for overnight. The solvent was removed under the reduced pressure, and the residue was diluted with CH_2Cl_2. The diluted solution was washed with brine, dried over $MgSO_4$, and concentrated in vacuo. The residue was purified by column chromatography on silica (EtOAc/*n*-hexane = 1/1 to 4/1). Compound **4** (10 mg, 28%) was obtained as a white solid. ^{1}H-NMR (400 MHz, DMSO-*d*$_6$) δ 11.99 (s, 1H), 10.25 (s, 1H), 8.60 (s, 1H), 7.99 (d, *J* = 8.4 Hz, 1H), 7.78 (dd, *J* = 8.5, 1.6 Hz, 1H), 7.46 (s, 1H), 6.84 (dd, *J* = 6.4, 2.0 Hz, 4H), 6.20 (s, 1H), 5.32 (m, 1H), 4.28 (s, 1H), 4.13 (t, *J* = 10.4 Hz, 2H), 3.90 (d, *J* = 6.3 Hz, 1H), 3.67 (d, *J* = 5.4 Hz, 1H), 2.67 (m, 1H), 2.05 – 1.93 (m, 3H) 1.40 (s, 1H); ^{13}C{^{1}H}-NMR (500 MHz, $CDCl_3$) δ 195.4, 170.9, 168.8, 168.6, 167.0, 165.7, 162.3, 162.1, 156.1, 145.1, 144.9, 141.2, 139.3, 137.1, 136.7, 132.7(2C), 132.2, 130.9(2C), 129.9(2C), 129.1(2C), 128.0, 126.5, 119.9, 117.8, 116.7, 114.4, 68.4, 66.8, 64.3, 31.8, 22.9, 20.4, 14.2, 6.6(2C); LR-MS (ESI$^+$) calculated for [M+H$^+$] 672.2; found 672.3.

N-Cyclopropyl-4′-(4-(2-(2-((2-(2,6-dioxopiperidin-3-yl)-1,3-dioxoisoindolin-4-yl)oxy)ethoxy)ethoxy)benzoyl)-6-methyl-[1,1′-biphenyl]-3-carboxamide (5)

Compound **5** (12 mg, 28%) was obtained as a white solid from **15b** (38 mg, 0.062 mmol) using the procedure similar to that for **4**. ^{1}H-NMR (400 MHz, DMSO-*d*$_6$) δ 11.10 (s, 1H), 8.41 (s, 1H), 8.29 (s, 1H), 7.73 (dd, *J* = 7.5, 2.1 Hz, 5H), 7.52 (t, *J* = 6.8 Hz, 3H), 7.44 – 7.34 (m, 2H), 7.58 – 7.50 (m, 2H), 7.07 (t, *J* = 8.4 Hz, 2H), 5.11 (m, 1H), 4.35 (s, 1H), 4.17 (dd, *J* = 17.9, 1.1 Hz, 2H), 3.92 – 3.79 (m, 3H), 3.73 (s, 1H), 3.48 (m, 1H), 3.53 – 3.43 (m, 1H), 2.95 (m, 1H), 2.83 – 2.49 (m, 2H), 2.27 (s, 1H), 2.16 – 1.87 (m, 2H), 0.65 (d, *J* = 4.8 Hz, 2H), 0.53 (s, 2H); ^{13}C{^{1}H}-NMR (500 MHz, $CDCl_3$) δ 195.5, 171.1, 168.7, 168.2, 167.2, 167.0, 165.8, 162.6, 156.4, 145.1, 141.2, 139.2, 137.1, 136.4, 133.8, 132.6(2C), 132.1, 130.9(2C), 130.3, 129.8(2C), 128.9(2C), 128.2, 119.4, 116.3, 114.1, 70.5, 69.8, 69.6, 68.8, 67.5, 29.7, 23.5, 20.6, 14.2, 6.8(2C); LR-MS (ESI$^+$) calculated for [M+H$^+$] 716.3; found 716.3.

N-Cyclopropyl-4′-(4-((5-((2-(2,6-dioxopiperidin-3-yl)-1,3-dioxoisoindolin-4-yl)oxy)pentyl)oxy)benzoyl)-6-methyl-[1,1′-biphenyl]-3-carboxamide (6)

Compound **6** (13 mg, 33%) was obtained as a white solid from **15c** (60 mg, 0.099 mmol) using the procedure similar to that for **4**. ^{1}H-NMR (400 MHz, DMSO-*d*$_6$) δ 8.45 (m, 1H), 7.80 – 7.73 (m, 4H), 7.65 – 7.59 (m, 4H), 7.57 – 7.51 (m, 3H), 7.41 (d, *J* = 8.9 Hz, 1H), 7.10 (dd, *J* = 8.7, 2.8 Hz, 1H), 5.24 (m, 1H), 4.24 (t, *J* = 5.8 Hz, 1H), 4.14 – 4.00 (m, 2H), 3.67 (m, 1H), 2.85 (m, 1H), 2.68 (m, 1H), 2.31 (s, 3H), 2.19 – 1.96 (m, 3H), 1.96 – 1.84 (m, 2H), 1.75 (m, 1H), 1.63 (m, 1H), 1.56 – 1.40 (m, 4H), 0.70 – 0.64 (m, 2H), 0.59 – 0.53 (m, 2H); ^{13}C{^{1}H}-NMR (500 MHz, $CDCl_3$) δ 195.3, 171.2, 168.8, 168.6, 167.2, 165.8, 162.9, 156.6, 144.7, 141.2, 139.4, 136.6, 134.0, 132.7(2C), 132.3, 130.8(2C), 129.9(2C), 129.0(2C), 128.6, 128.1, 126.3, 119.1, 117.3, 115.9, 114.1, 69.3, 68.2, 62.9, 32.4, 31.0, 29.8, 28.9, 23.2, 22.5, 20.5, 6.9(2C); LR-MS (ESI$^+$) calculated for [M+H$^+$] 714.3; found 714.3.

N-Cyclopropyl-4′-(4-(2-(2-(2-((2-(2,6-dioxopiperidin-3-yl)-1,3-dioxoisoindolin-4-yl)oxy)ethoxy)ethoxy)ethoxy)benzoyl)-6-methyl-[1,1′-biphenyl]-3-carboxamide (7)

Compound **7** (11 mg, 29%) was obtained as a white solid from **15d** (67 mg, 0.101 mmol) using the procedure similar to that for **4**. ^{1}H-NMR (400 MHz, DMSO-d_6) δ 8.47 (s, 1H), 7.76 (d, J = 9.4 Hz, 7H), 7.55 (d, J = 7.3 Hz, 3H), 7.41 (m, 1H), 7.15 – 7.06 (m, 2H), 5.17(m, 1H), 4.33 (s, 1H), 4.19 (s, 2H), 3.77 (s, 2H), 3.70 – 3.48 (m, 5H), 3.42 (m, 1H), 2.97 (m, 1H), 2.88 – 2.66 (m, 1H), 2.54 (s, 1H), 2.30 (s, 1H), 2.00 (m, 1H), 0.68 (d, J = 5.2 Hz, 2H), 0.56 (s, 2H); ^{13}C{^{1}H}-NMR (500 MHz, $CDCl_3$) δ 195.5, 171.1, 168.4, 167.2, 165.8, 162.7, 156.4, 144.9, 141.3, 139.3, 136.9, 136.4, 133.9, 132.6(2C), 132.2, 130.9(2C), 129.8(2C), 129.0(2C), 128.2, 126.4, 119.6, 117.5, 116.2, 114.3(2C), 72.7, 70.8, 70.5, 69.6, 69.2, 67.8, 61.8, 29.8, 23.4, 21.9, 20.6, 6.9(2C); LR-MS (ESI^{+}) calculated for [M+H^{+}] 760.3; found 760.2.

2.2. Synthesis of Compound 14 and 16

2-(2-(4-(5′-(Cyclopropylcarbamoyl)-2′-methyl-[1,1′-biphenyl]-4-carbonyl)phenoxy)ethoxy)ethyl 5-((3*S*,4*S*,6*R*)-2-oxohexahydro-1*H*-thieno[3,4-*d*]imidazol-4-yl)pentanoate (14)

To a solution of **8b** (28 mg, 0.061 mmol, 1 eq), biotin (14.9 mg, 1 eq), and DMF (0.4 mL) was added EDC (11 mg, 1.2 eq) and 4-dimethylaminopyridine (0.9 mg, 0.12 eq) under Ar at 0 °C. Then, the reaction mixture was warmed to room temperature and stirred for 3 days. After removing the solvent under the reduced pressure, the residue was purified by flash column chromatography on silica (MeOH/EtOAc = 9/1). **14** (17.7 mg, 42%, 79% for brsm) was obtained as a white solid. ^{1}H-NMR (400 MHz, $CDCl_3$) δ 7.84 (dd, J = 18.5, 8.5 Hz, 4H), 7.68 (dd, J = 7.9, 1.9 Hz, 1H), 7.64 (s, 1H), 7.42 (d, J = 8.2 Hz, 2H), 7.34 (d, J = 7.9 Hz, 1H), 7.02 (d, J = 8.8 Hz, 2H), 6.40 (s, 1H), 5.36 (s, 1H), 4.93 (s, 1H), 4.47 (t, J = 6.1 Hz, 1H), 4.31 – 4.21 (m, 5H), 3.91 (t, J = 4.6 Hz, 2H), 3.79 (t, J = 4.7 Hz, 2H), 3.13 (q, J = 6.8, 6.3 Hz, 1H), 2.91 (d, J = 6.2 Hz, 3H), 2.71 (d, J = 12.8 Hz, 1H), 2.34 (d, J = 11.7 Hz, 5H), 1.71 – 1.62 (m, 7H), 1.49 – 1.38 (m, 2H), 1.26 (s, 7H), 0.90 – 0.83 (m, 3H), 0.67 – 0.58 (m, 2H); LR-MS (ESI$^+$) calculated for [M+H$^+$] 686.3; found 686.4.

2-(2-Phenoxyethoxy)ethanol (15)

A solution of phenol (376mg, 4 mmol, 2 eq), K_2CO_3 (1106 mg, 6 eq), DMF (5 mL), and acetone (10 mL) was stirred and refluxed for 1 h. After cooling to room temperature, 2-(2-chloroethoxy)ethanol (249 mg, 2 mmol, 1 eq) and NaI (300 mg, 1 eq) was added. The reaction mixture was refluxed for overnight. After cooling, the mixture was quenched with 2 N HCl aqueous solution and extracted with EtOAc. The combined organic layer was dried over $MgSO_4$ and concentrated in vacuo. The residue was purified by column chromatography on silica (EtOAc/*n*-hexane = 1/2). Compound **15** (220 mg, 60%) was obtained as a colorless oil. ^{1}H-NMR (400 MHz, $CDCl_3$) δ 7.33 – 7.27 (m, 2H), 7.00 – 6.90 (m, 3H), 4.18 – 4.11 (m, 2H), 3.92 – 3.85 (m, 2H), 3.78 – 3.75 (m, 2H), 3.70 – 3.66 (m, 2H).

2-(2-Phenoxyethoxy)ethyl 5-((3*S*,4*S*,6*R*)-2-oxohexahydro-1*H*-thieno[3,4-*d*]imidazol-4-yl)pentanoate (16)

Compound **16** (33.8 mg, 45%) was obtained as a white solid from compound **15** (33.6 mg, 0.184 mmol) using the procedure similar to that for **14**. ^{1}H-NMR (400 MHz, $CDCl_3$) δ 7.32 – 7.27 (m, 2H), 7.00 – 6.88 (m, 3H), 5.59 (s, 1H), 5.02 (s, 1H), 4.49 – 4.42 (m, 1H), 4.29 – 4.23 (m, 3H), 4.17 – 4.10 (m, 2H), 3.90 – 3.83 (m, 2H), 3.78 (t, J = 4.7 Hz, 2H), 3.17 – 3.08 (m, 1H), 2.88 (dd, J = 12.8, 5.0 Hz, 1H), 2.72 (d, J = 12.8 Hz, 1H), 2.37 (t, J = 7.3 Hz, 2H), 1.70 – 1.63 (m, 5H), 1.47 – 1.41 (m, 2H); LR-MS (ESI$^+$) calculated for [M+H$^+$] 409.2; found 409.2.

3. Biological Assay

Cells culture and plasmids. Huh7 cell line was purchased from KCLB (Korea cell line bank). BV-2, CD-D1a, HT-22 and N2a cells were purchased from ATCC. Cells were cultured in Dulbecco's modified Eagle's medium (DMEM) containing 10% fetal bovine serum (Hyclone), 100 U/mL penicillin, and 100 μg/mL streptomycin (Hyclone). pCDNA3 Flag p38α WT and pCDNA3 Flag p38α T180A/Y182F (agf) mutant plasmids were purchased from addgene. p38α D176A mutant were inserted into pCDNA3 vector with a *N*-terminus Flag tag. p38α WT, p38α D176A mutant and p38α T180A/Y182F mutant were cloned into pMT HA vector. The P301L/S320F human tau construct were cloned into pIRES puro3 vector. The PCR-mediated site-directed mutagenesis was confirmed by sequencing.

Transfection. For ectopic expression in HEK293T cells, conventional Calcium-phosphate transfection (calcium chloride, 2X HEPES Buffered Saline (HBS)) methods were conducted. HEK293T cells were seed in cell culture dishes. Plasmid DNA in distilled water were mixed with 2M Calcium chloride. The mixture was mixed evenly using a vortex mixer and placed in 2X HBS. The final mixture was incubated for 10 minutes and then treated with prepared HEK293T cells. HEK293T cells were cultured in CO_2 incubation for more than 12 h to allow for over expression before use in experiments.

Biotin-Pulldown assay. Cells were lysed using 0.1% TritonX-100. The compound synthesized with biotin is added to the lysate and reacted at 37 °C for 2h. Then, Streptavidin-coated resin was added to the lysate and reacted at 37 °C for 0.5h. After washing with 0.1% Triton X-100 lysis buffer, 2X sample buffer was added and boiled at 100 °C for 10 minutes. Protein samples were analyzed by SDS-PAGE and Immunoblotting.

Immunoblotting and Immunoprecipitation. Cells and male mice brain were lysed in RIPA buffer supplemented with 100X protease/phosphatase inhibitor cocktail. Lysates were centrifuged at 13,000 × g, the supernatants were used for SDS-PAGE. Immunoblotting was conducted with standard manual. For immunoprecipitation assay, cells were lysed with 0.5% triton X-100 buffer supplemented with 2 mM NaF, 1 mM Na_3VO_4, and 100X protease/phosphatase inhibitor cocktail. The supernatants were incubated with following antibodies at 4 °C overnight. Protein A / G resin (Sigma-Aldrich) was added and reacted at 4 °C for 4 h. After vigorous washing with 0.5% triton X-100 lysis buffer, 5X sample buffer was added and boiled at 100 °C for 10 minutes for sampling. Protein samples were analyzed by SDS-PAGE and Immunoblotting.

RT-qPCR. Total RNA from cells and male mice brain were isolated using RNA isolation and preparation kit according to the instructions of the manufacture's. The concentration and purity of isolated total RNAs measured with a spectrophotometer. 1000 ng of total RNA was synthesized to cDNA. Synthesized cDNA was subjected to qRT-PCR with SYBR Green Mix, using CFX connect real-time PCR system. The primers are listed in Table S2.

Separation of insoluble fraction. The cells and brains were lysed using 1% triton X-100 buffer and the supernatant was centrifuged at 13,000 × g for 30min at 4°C. The supernatants were retained and regarded as the soluble S1 fraction. The resulting pellets were then resuspended in SDS buffer (10 mM Tris-HCl, pH 7.4, 150 mM NaCl, 1% SDS) and centrifuged for 30min

at 13,000 × g (4°C). The supernatants were retained and regarded as the insoluble S2 fraction. S1 and S2 fractions were diluted in 5× Sample buffer, and boiled for 10 min, separated by SDS-PAGE, and analyzed by immunoblotting as described.

ELISA. An enzyme-linked immunosorbent assay (ELISA) for human Aβ1–40 and Aβ1–42 was performed using fluorescence-based ELISA kits (Invitrogen) and appropriate Aβ standards in compliance with the manufacturer's protocol. The hippocampus and frontal cortex from one hemisphere of male mice were homogenized in 10X volume of guanidine buffer with a final concentration of 50 mM Tris and 5 M guanidine HCl at pH 8.0. Homogenates were mixed at room temperature for 4 h and then diluted in PBS containing 5% BSA, 0.03% Tween 20, and protease inhibitor cocktail. The protein levels of IL-1β and IL-12 in BV-2 cells were detected by ELISA assay according to manufacturer's instructions, respectively (R&D Systems and Abfrontier). The protein levels of IL-6 and TNF-α in BV-2 cells were detected by Mouse Magnetic Luminex immunoassay according to manufacturer's instructions, respectively (R&D Systems).

Animal and treatment. 5xFAD mice (34830-JAX, Tg6799) were purchased and maintained in an individual ventilated cage with 12 h light/dark cycles at 22 °C. Similar to previous studies,[2] 8-months-old 5xFAD mice were divided into two groups (vehicle vs. treatment group). Treatment group was treated with **2**, 8 μg a day, three times a week for 5-weeks. Meanwhile, vehicle group and their wild-type littermates were treated with the same volume of vehicle. **2** was dissolved in 50% DMSO, 50% tween 20 at 40 mg/mL which was used as a 40X stock solution. To prepare a working solution, an aliquot of the 40x stock solution was diluted in autoclaved saline. The **2** working solution and vehicle were treated to the mice by intranasal delivery using the Jelly chair system.[3] For every intranasal administration, all mice were anesthetized by 50 mg/kg Ketamine, 25 mg/kg Xylazine solution. Both male and female mice were used in the behavior test. But, to minimize the sex difference on the degree of amyloidopathy, the protein and RNA samples were obtained from male mice. And the histological sections were acquired from female mice. All experiments were approved by the Kyung Hee University Institutional Animal Care and Use Committee (IACUC, KHUASP(SE)-19-035).

MAPT-P301S mutation mice (PS19) and their wild-type background mice were purchased from Jackson Laboratory (Stock No: 008169). The mice were bred and maintained in individual ventilated cages with 12-h light/dark cycles. PS19 mice were expressing the human microtubule-associated protein tau (*MAPT*) gene with a P301S mutation under the mouse prion protein promoter (*Prnp*). The 6-months PS19 mice were divided into two groups (vehicle vs. treatment group). PRZ-18002 or vehicle were treated to each group for 5-weeks in the same manner as 5xFAD mouse. Both male and female mice were used in the behavior test, and only male mice were used in protein analysis. All experiments were approved by the Kyung Hee University Institutional Animal Care and Use Committee (IACUC, KHUASP-20-231)

Brain distribution measurement. ICR mice were treated with PRZ-18002 at 8 μg/8 μl by intranasal administration. The mouse brains were dissected after 5-, 15-, 30-min and 1-, 2-, 8-hour of single treatment. The brains were stored at -80℃ until analysis. For bioanalysis, the brain samples were prepared by homogenizing the whole brains at 3X volume of methanol, and the PRZ-18002 concentration were measured by Applied Biosystems MDS SCIEX API 4000 Triple Quadrupole Mass Spectrometer (SCIEX, Framingham, MA, USA) with an ESI source in the positive ion mode. The final concentrations of the calibration standards were 1,

2, 5, 10, 20, 50, and 100 ng/mL, and those of the QC samples were 3, 15, and 80 ng/mL. All the analytical procedures were conducted according to the guidelines of the bioanalytical method, the Korean Ministry of Food and Drug Safety guidelines.

Behavior test. The Morris water maze task was performed as previously described.[2] In brief, the mice were habituated to water and swimming, in the absence of the cues, one day before training period. In the training period, the cues were displayed around the maze. The mice swam in the water maze until arriving the hidden platform within maximum 60 seconds and the arrived times were recorded. At day 11, the hidden platform was removed, and the probe task were performed for 60 seconds. All trials were recorded using a camera and the video images were analyzed using the free tracking software, Toxtrac (https://toxtrac.sourceforge.io). For passive avoidance tests (PAT), mice were dark-adapted for 30-min and placed at the bright compartment of the PAT chamber. Then the latency times when the mice entered into the dark compartment were recorded and they were received a mild foot shock (Day-1). The same procedure was repeated without foot shock at Day-2, with a maximum latency of 5-min.

Tissue preparation and immunofluorescence. For tissue preparation, female mice were anesthetized with a Ketamine-Xylazine mixed solution and perfused through the heart with PBS followed by 4% paraformaldehyde (PFA) solution. Mouse brains were obtained and further fixed within 4% PFA at 4 ^{o}C overnight, then the brains were incubated in 30% sucrose solution. To make cryoblocks, the brain samples were embedded in O.C.T. compound and frozen at -80 ^{o}C. Using a cryostat (CM30 50S; Leica), sequential 30-μm coronal sections were obtained. For immunostaining, the brain sections were blocked with 2% normal goat serum, 2% BSA and 0.4% Triton-X100; followed by incubation with primary antibodies at 4 ^{o}C overnight. After that, the sections were incubated with host-matched secondary antibodies conjugated with Alexa-Fluorescence.

Confocal microscopy and image analysis. Immunostained images were obtained using confocal microscopy (Z-stack, 1.5-μm interval, 15 optical slices). Four cortex area and four hippocampus area were imaged and used for analyzing one brain slice. To quantifying one mouse, six brain slices were used (Bregma -1.4 to -2.5). The images were analyzed using Image J software.

Statistics. All values are expressed as the mean ± SEM. Results were analyzed using the Student's t test or analysis of variance (ANOVA) for multiple comparisons. In all *in vivo* studies, n means the number of animals used in the corresponding data. All data was analyzed using SPSS.25 with statistical significance at $P < 0.05$. Parametric analysis was used if the data satisfied with the null hypothesis of the Levene's test. Morris water maze latency was analyzed using the generalized estimating equation (GEE) analysis. All graphs were constructed using Graph Pad Prism 5.0 software and all data were represented in mean ± standard error of mean (SEM).

4. Molecular Modeling Studies

The modeling tasks were conducted by Schrödinger Suite (2021-3 release, Schrödinger, LLC, NY, USA), and the molecular graphic figures were generated using PyMOL v.2.5.2 (Schrödinger, LLC, NY, USA). All computational tasks were performed on a Linux CentOS 7.7 workstation with Intel® Xeon® GOLD 36-core 3.1 GHz processor.

Protein and ligand preparation. The X-ray crystal structures of p-p38α (PDB id: 6ZQS)[4] and CRBN-DDB1-CK1 complex (PDB id: 5FQD)[5] were prepared by using Protein Preparation Wizard in Maestro, version 13.1 (Schrödinger, LLC, NY, USA). Bond orders were assigned, hydrogen atoms were added, and protonation states of the residues at pH 7.4 were generated by Epik (Schrödinger, LLC, NY, USA). All hydrogen atoms were energy-minimized with the optimized potential for liquid simulation (OPLS) 4 force field until the average root-mean-square deviation for hydrogen atoms reached 0.30 Å. Ligand molecules were prepared by LigPrep module in Schrödinger. The generation of ionization states significantly populated in pH 7.4 was performed with Ionizer. The geometries of the generated ligand structures were energy-minimized using OPLS4 force field, while keeping the chirality from the input files throughout the calculation.

Induced-fit docking. To fully consider the flexibility of both the ligand and the receptor in the docking study, we adopted the induced-fit docking (IFD) protocol implemented in Schrödinger. It is based on Glide docking and the refinement module in Prime that accurately predicts the ligand binding modes and concomitant structural changes in the receptor. In this study, the center of the grid box was defined by the co-crystal ligand of each input PDB, and all docking calculations were run in the Standard Precision (SP) mode of Glide. The residues within 5 Å from the docked conformations were refined by Prime module.

Complex structure modeling and optimization. By using the docked conformation of PRZ-18002$^{\text{p38 ligand}}$ in p-p38α, the docked conformation of PRZ-18002$^{S\text{-pomalidomide}}$ in CRBN, and the geometry of CK1$^{\text{kinase domain}}$-CRBN-DDB1 complex structure, the quaternary complex model of PRZ-18002, p-p38α, CRBN, and DDB1 was constructed. The resulting structure was energy-minimized with the distance constraint of ~8 Å between PRZ-18002$^{\text{p38 ligand}}$ and PRZ-18002$^{S\text{-pomalidomide}}$, and the linker moiety was manually modeled by connecting the two ligands. Further energy minimization (without any constraint) was performed to obtain the final complex model (Fig. 2E).

5. Supplementary Tables

5.1. Table S1. Information of antibodies used in this study

Target	Host	Source	Catalog no.	RRID	Application
phospho-p38	Rabbit	Cell Signaling Technology	9215	AB_331762	WB / 1:1000
phospho-p38	Rabbit	Cell Signaling Technology	9211	AB_331641	WB / 1:1000<br>IP/ 1:100
p38	Rabbit	Cell Signaling Technology	9212	AB_330713	WB / 1:1000

HA-Tag	Mouse	Cell Signaling Technology	2367	AB_10691311	WB / 1:1000
Flag-Tag	Rabbit	Cell Signaling Technology	2368	AB_2217020	WB / 1:1000
β-actin (HRP)	Mouse	Santa Cruz Biotechnology	sc-47778	AB_2714189	WB / 1:5000
p-MKK3	Rabbit	Cell Signaling Technology	9231	AB_2140799	WB / 1:1000
MKK3	Rabbit	Cell Signaling Technology	5674	AB_10828087	WB / 1:1000
p-MKK4	Rabbit	Cell Signaling Technology	4514	AB_2140946	WB / 1:1000
MKK4	Rabbit	Cell Signaling Technology	9152	AB_330905	WB / 1:1000
p-MK2	Rabbit	Cell Signaling Technology	3007	AB_490936	WB / 1:1000
MK2	Rabbit	Cell Signaling Technology	3042	AB_10694238	WB / 1:1000
p-HSP27	Rabbit	Cell Signaling Technology	9709	AB_11217429	WB / 1:1000
HSP27	Rabbit	Cell Signaling Technology	2442	AB_2233273	WB / 1:1000
P4D1(Ub)	Mouse	Cell Signaling Technology	3936	AB_331292	WB / 1:1000
P38α	Rabbit	Cell Signaling Technology	9218	AB_10694846	WB / 1:1000
P38β	Rabbit	Cell Signaling Technology	2339	AB_823587	WB / 1:1000
P38γ	Rabbit	Cell Signaling Technology	2307	AB_659929	WB / 1:1000
P38δ	Rabbit	Cell Signaling Technology	2308	AB_10694398	WB / 1:1000
p-MEK	Rabbit	Cell Signaling Technology	9217	AB_331298	WB / 1:1000
p-JNK	Rabbit	Cell Signaling Technology	4668	AB_823588	WB / 1:1000
p-ERK	Rabbit	Cell Signaling Technology	4370	AB_2315112	WB / 1:1000
p-tau (Ser396)	Rabbit	Abcam	ab109390	AB_297597	WB / 1:1000
Tau (tau-5)	Rabbit	Thremo	AHB0042	AB_2536235	WB / 1:1000
p-tau (AT8)	Mouse	Invitrogen	MN1020	AB_223647	WB / 1:500
p-tau (Thr231)	Mouse	Invitrogen	MN1040	AB_223649	WB / 1:1000
Tau (HT7)	Mouse	Invitrogen	MN1000	AB_2314654	WB / 1:1000
Human β-amyloid 1-16 (clone: 6E10)	Mouse	BioLegend	803001	AB_2564653	WB / 1:1000<br>IHC / 1:500
Iba-1	Rabbit	Wako	019-19741	AB_839504	IHC / 1:500
GFAP	Chicken	Abcam	ab4674	AB_304558	IHC / 1:500
β-actin (HRP)	Mouse	Santa Cruz Biotechnology	sc-47778 HRP	AB_2714189	WB / 1:5000
Mouse IgG (HRP)	Goat	Santa Cruz Biotechnology	sc-2005	AB_631736	WB / 1:5000

Rabbit IgG (HRP)	Goat	Santa Cruz Biotechnology	sc-2054	AB_631748	WB / 1:5000
Rabbit IgG (488)	Goat	Invitrogen	A11008	AB_143165	IHC / 1:1000
Rabbit IgG (594)	Goat	Invitrogen	A11012	AB_2534079	IHC / 1:1000
Mouse IgG (594)	Goat	Invitrogen	A11005	AB_2534073	IHC / 1:1000
Chicken IgY (488)	Goat	Invitrogen	A11039	AB_2534096	IHC / 1:1000

WB, Western blot; IHC, Immunohistochemistry.

5.2. Table S2. Information of qRT-PCR primers used in this study

Gene name	NCBI reference	Forward primer (5’→3’)	Reverse primer (5’→3’)
TNF-α	NM_013693	GATTATGGCTCAGGGTCCAA	GCTCCAGTGAATTCGGAAAG
IL-1β	NM_008361	CCCAAGCAATACCCAAAGAA	GCTTGTGCTCTGCTTGTGAG
IL-6	NM_031168	CCGGAGAGGAGACTTCACAG	TTGCCATTGCACAACTCTTT
IL-12	NM_001303244	GCTTCTCCCACAGGAGGTTT	CTAGACAAGGGCATGCTGGT
β-actin	NM_007393	TGGAATCCTGTGGCATCCAT	TAAAACGCAGCTCAGTAACA

6. Supplementary Figures

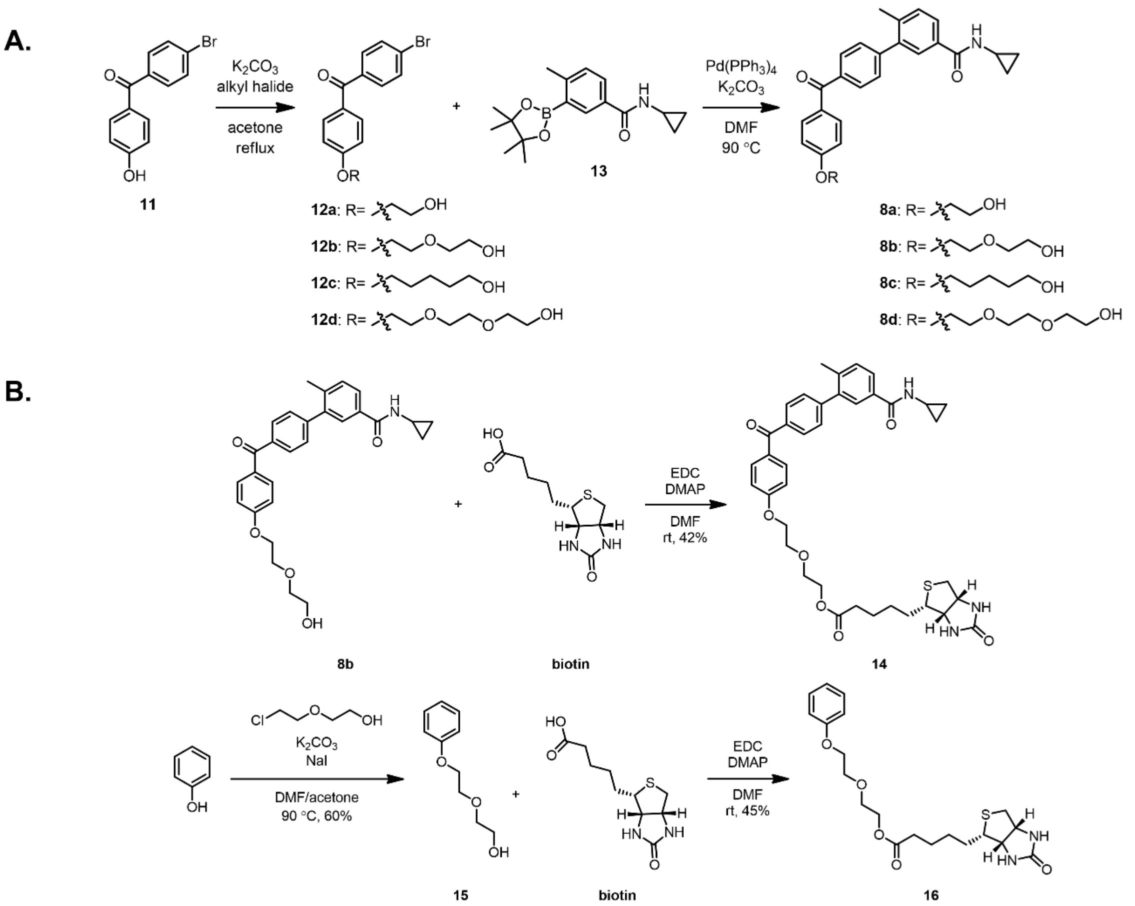

Figure S1. (A) Synthetic Scheme of p38 ligands **8a-d** from known compound **11**. (B) Synthetic Scheme of compound **14** and **16**.

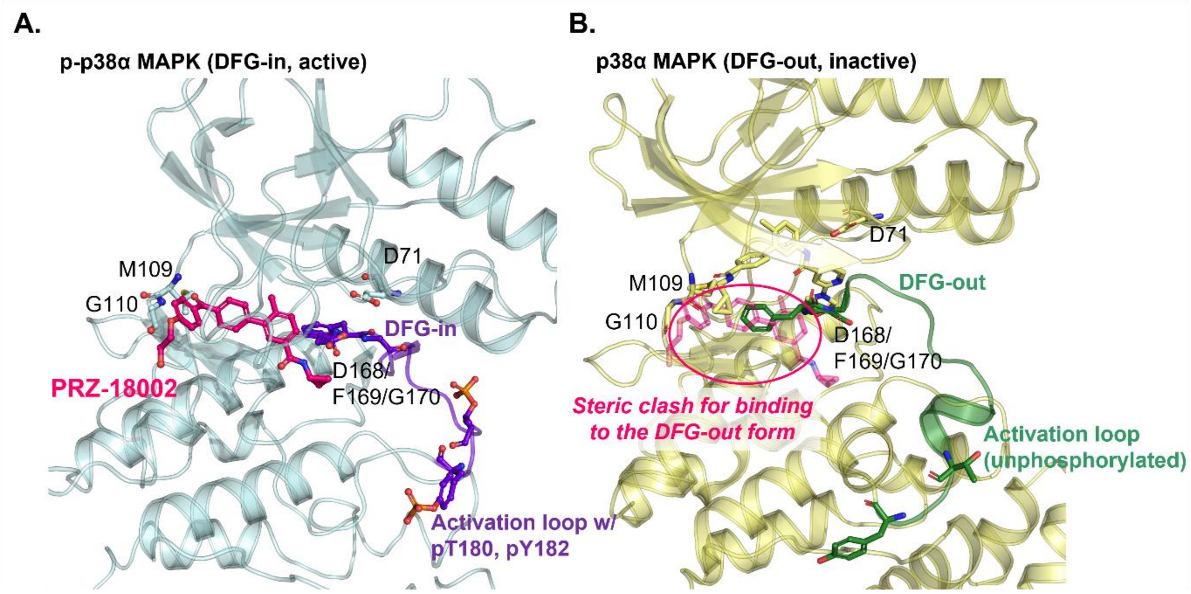

Figure S2. PRZ-18002 preferably binds to p-p38. (A) Docked mode of the p38 ligand part of PRZ-18002 in the active p-p38α with DFG-in conformation (PDB id: 6ZQS)[4]. The bound ligand is represented in sticks with the carbon atom in magenta. The DFG motif and the activation loop of p-p38 are colored in purple. The hinge region (M109, G110), DFG motif (D168, F169, G170), and the phosphorylated T180 and Y182 are depicted in sticks. (B) Structure of the inactive p38α with DFG-out conformation (PDB id: 3D83)[6]. The DFG motif and the activation loop (unphosphorylated) are colored in green. The docked conformation of p38 ligand part of PRZ-18002 in p-p38α is overlaid and represented by transparent magenta sticks.

A.

Kinase Tested	[ATP] Tested	IC50	Hillslope	R^2 Value	Conc of Compound	% Inhibition		Development Reaction Interference	Test Compound Interference		Z'
	(µM)	(nM)			(nM)	Point 1	Point 2		Coumarin	Fluorescein	
MAPK14 (p38 alpha) Direct	Km app	135	1.48	0.9959	1000	92	97	Pass	Pass	Pass	0.70
MAPK14 (p38 alpha) Direct	Km app	135	1.48	0.9959	333	85	76	Pass	Pass	Pass	0.70
MAPK14 (p38 alpha) Direct	Km app	135	1.48	0.9959	111	43	39	Pass	Pass	Pass	0.70
MAPK14 (p38 alpha) Direct	Km app	135	1.48	0.9959	37.0	12	19	Pass	Pass	Pass	0.70
MAPK14 (p38 alpha) Direct	Km app	135	1.48	0.9959	12.3	1	1	Pass	Pass	Pass	0.70
MAPK14 (p38 alpha) Direct	Km app	135	1.48	0.9959	4.12	-2	-8	Pass	Pass	Pass	0.70
MAPK14 (p38 alpha) Direct	Km app	135	1.48	0.9959	1.37	2	2	Pass	Pass	Pass	0.70
MAPK14 (p38 alpha) Direct	Km app	135	1.48	0.9959	0.457	0	1	Pass	Pass	Pass	0.70
MAPK14 (p38 alpha) Direct	Km app	135	1.48	0.9959	0.152	2	-1	Pass	Pass	Pass	0.70
MAPK14 (p38 alpha) Direct	Km app	135	1.48	0.9959	0.0495	2	2	Pass	Pass	Pass	0.70

10-point titration results of compound **2**

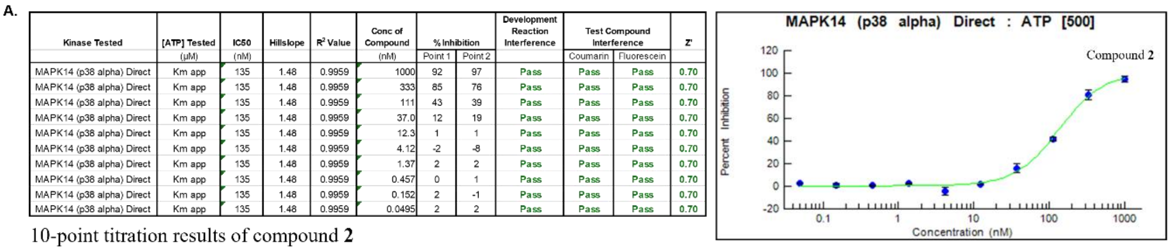

Standard curve analysis for determination of IC_{50} of compound **2** (IC_{50} = 135 nM)

B.

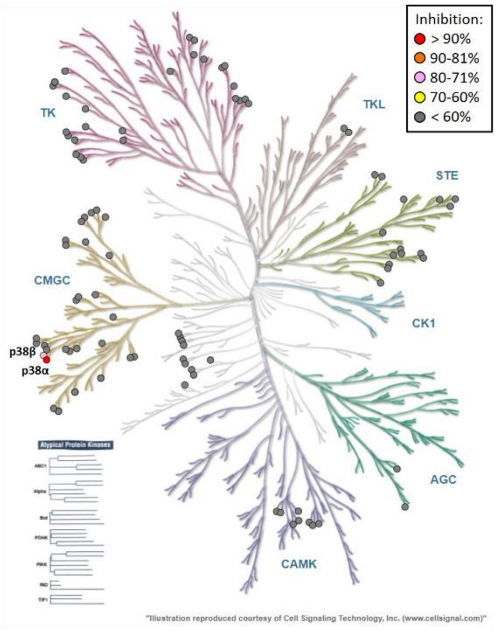

C.

Kinase	% activity remaining	Kinase	% activity remaining	Kinase	% activity remaining[b]	Kinase	% activity remaining
ABL1	97	DYRK2	113	JAK2	99	MNK1	108
ABL2	95	DYRK3	98	JAK3	103	MNK2	106
BLK	94	DYRK4	100	KDR (VEGFR2)	93	MST1R	92
BTK	96	EGFR (ErbB1)	101	LCK	80	MST4	95
BRAF	94	ERBB2 (HER2)	111	LTN A	93	NEK6	99
CAMK2α	98	ERBB4 (HER4)	98	LYN B	86	PAK1	85
CAMK2β	83	FLT1 (VEGFR1)	95	MAPK1 (ERK2)	99	PAK2	106
CAMK2δ	97	FLT4 (VEGFR3)	95	MAPK3 (ERK1)	98	PAK3	97
CAMKK1	106	FLT3	94	MAPK8 (JNK1)	90	PAK4	95
CAMKK2	107	FRK (PTK5)	92	MAPK9 (JNK2)	97	PAK7	100
CDC42BPA	95	GSK3α	93	MAPK10 (JNK3)	96	PTK6	93
CDK1/cyclin B	94	GSK3β	97	**MAPK14 (p38α) direct**	**9**	RAF1 Y340D Y341D	90
CDK2/cyclin A	100	HCK	98	**MAPK11 (p38β)**	**22**	ROCK2	99
CDK3/cyclin E1	102	HIPK1	98	MAPK12 (p38γ)	101	SLK	102
CDK5/p25	101	HIPK2	97	MAPK13 (p38δ)	97	SRC	90
CDK5/p35	100	HIPK3	100	MAPK15 (ERK7)	99	STK3	100
CDK7/cyclin H/MNAT1	101	HIPK4	97	MAPKAPK2	92	STK24	101
CDK8/cyclin C	103	IIPK1	102	MAPKAPK3	95	TEC	102
CDK9/cyclin K	100	ICK	110	MAPKAPK5	100	TBK1	97
CDK9/cyclin T1	112	IKK α	98	MEK1	94	TXK	97
CDK11/cyclin C	106	IKK β	97	MEK2	100	TYK2	93
CSK	100	IKK ε	99	MEKK2	108	ULK1	96
DYRK1A	102	ITK	94	MEKK3	107	ULK2	106
DYRK1B	98	JAK1	98	MKK6	103	ULK3	101

Kinase selectivity of synthesized compound (1 µM) was profiled through SelectScreen™ Kinase Profiling Services (Life technologies). Data are given in percent remaining activity (i.e., 100% indicates no observed inhibition).

Figure S3. PRZ-18002 inhibited p-p38 in dose dependent manner and showed significant selectivity towards p38 among kinases. (A) (B) and (C) Kinase inhibition activities of synthesized PRZ-18002 were tested through SelectScreen™ Kinase Profiling Services (Life technologies). Enzymatic assays were performed at an ATP concentration corresponding to Km app or 100 mM, and each point was determined in duplicate experiments. IC_{50} of the compound was determined in 10-point titration.

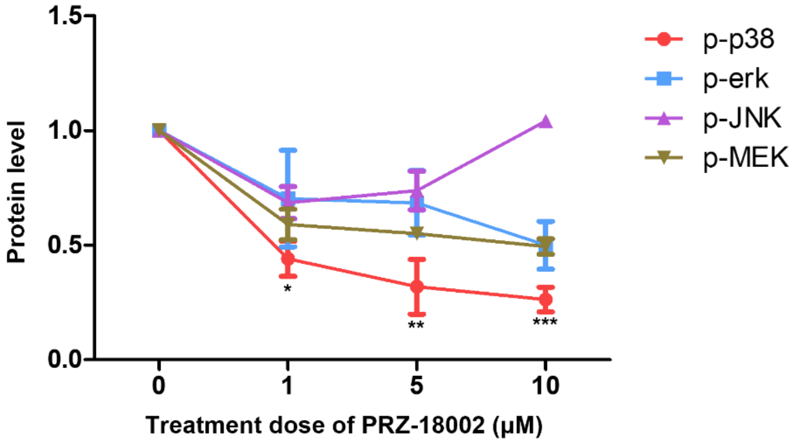

Figure S4. PRZ-18002 induces degradation of phospho-p38 protein but does not other MAPKs such as phospho-ERK, phospho-JNK and phospho-MEK. Huh7 cells were treated with **2** at the concentrations shown in the figure. Cell lysates were analyzed with anti-p-p38 antibody, anti-p-ERK, anti-p-JNK and anti-p-MEK antibody. The graph show quantification of indicated protein levels. The western blot is representative of three independent experiments (n=3). Data are shown as mean ± SEM(*$P < 0.05$; **$P < 0.01$; ***$P < 0.001$ vs. control group).

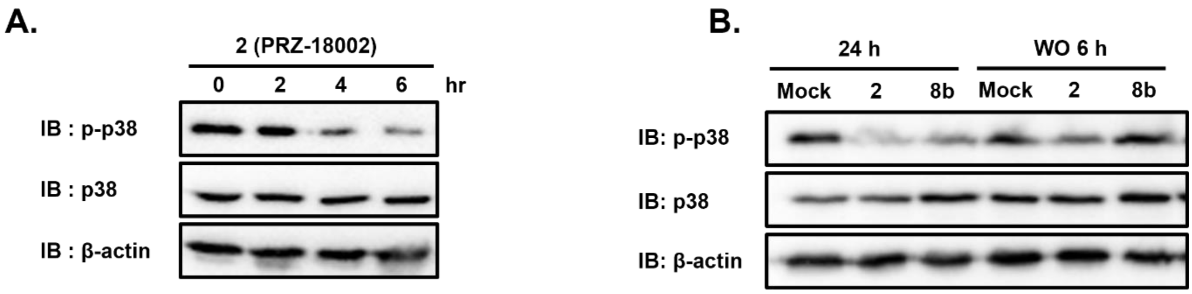

Figure S5. The decrease in the protein levels of p-p38 by PRZ-18002 is time-dependent and the effect persists even after drug removal. (A) PRZ-18002-mediated degradation was in a time dependent manner. Huh7 cells were incubated with 10 μM **2** for the indicated times. Cell lysates were analyzed by immunoblotting. (B) A decrease in the p-p38 protein was analyzed by removing the **2** or **8b** after treatment. Huh7 cells were treated with 10 μM **2** or **8b**. Thereafter, the media was removed after 6 h, replaced with a new media without drugs, and incubated for 18 h. Cell lysates were analyzed by immunoblotting using anti-p-p38 antibody, anti-p38 antibody and anti-β-actin antibody.

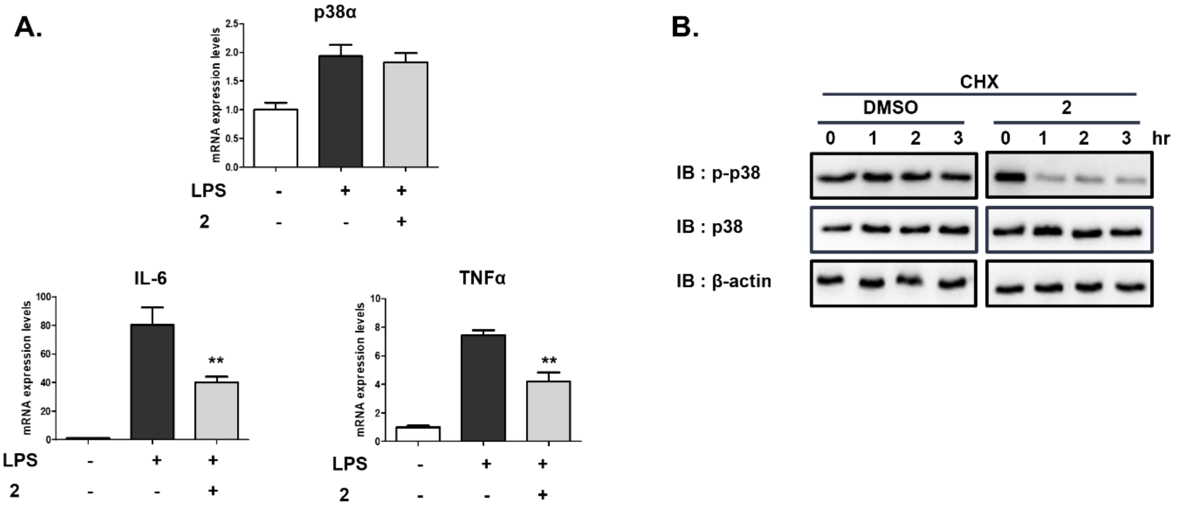

Figure S6. The decrease in the amount of p38 protein independent of the p38 mRNA levels by PRZ-18002 induces a decrease in the amount of mRNA of inflammatory cytokines. (A) BV-2 cells were treated with 10 μM **2** together with 1 μg/mL LPS. Then cells were harvested after incubation for 24 h. mRNA levels were measured by RT-PCR and the graph show quantification of mRNA levels of indicated protein. Data are shown as mean ± SEM (**P < 0.01 vs. LPS group). (B) Huh7 cells were treated with DMSO or 10 μM **2** together with 10 μg/mL CHX for the indicated times. Cell lysates were analyzed with antibodies indicated in the figure.

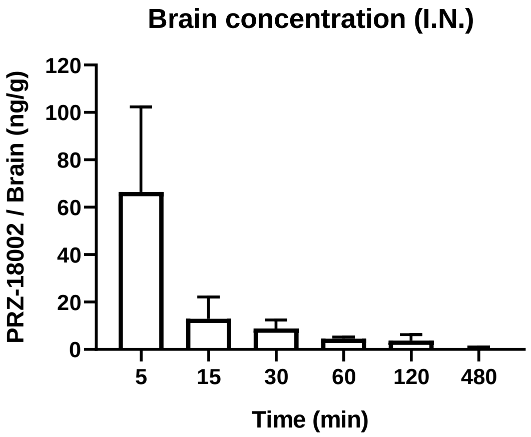

Figure S7. Brain concentration-time profile of PRZ-18002 after single 8 μg intranasal administration. Mice were treated with PRZ-18002 at 8 μg/8 μl by intranasal administration. Mouse brains were dissected after 5, 15, 30 min and 1, 2, 8 hour of single treatment. Data are shown as mean ± S.D. (n = 3 per time points).

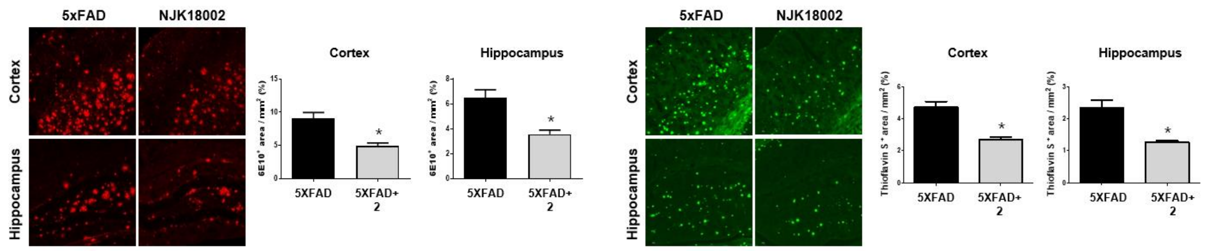

Figure S8. **PRZ-18002 reduced Aβ deposits in 9-months-old 5xFAD mice.** (A and B) Representative confocal microscope images and quantification data of Aβ deposits stained with 6E10 antibody (A) and thioflavin S (B). 6E10- and thioflavin S-positive areas per square millimeter were quantified. For quantification, four areas in each section and six sections in each mouse were used. Data are shown as mean ± SEM (Scale bar, 100 μm, n = 5 per group, Student's t-test, *P < 0.05 vs. vehicle-treated 5xFAD).

7. References

1. Heo, J.; Shin, H.; Lee, J.; Kim, T.; Inn, K.-S.; Kim, N.-J., Synthesis and biological evaluation of N-cyclopropylbenzamide-benzophenone hybrids as novel and selective p38 mitogen activated protein kinase (MAPK) inhibitors. *Bioorg. Med. Chem. Lett.* **2015,** *25*, 3694-3698.
2. Gee, M. S.; Son, S. H.; Jeon, S. H.; Do, J.; Kim, N.; Ju, Y.-J.; Lee, S. J.; Chung, E. K.; Inn, K.-S.; Kim, N.-J.; Lee, J. K., A selective p38α/β MAPK inhibitor alleviates neuropathology and cognitive impairment, and modulates microglia function in 5XFAD mouse. *Alzheimer's Res. Ther.* **2020,** *12*, 45.
3. Ullah, I.; Chung, K.; Beloor, J.; Lee, S.-K.; Kumar, P., A Positioning Device for the Placement of Mice During Intranasal siRNA Delivery to the Central Nervous System. *JoVE* **2019**, e59201.
4. Kirsch, K.; Zeke, A.; Tőke, O.; Sok, P.; Sethi, A.; Sebő, A.; Kumar, G. S.; Egri, P.; Póti, Á. L.; Gooley, P.; Peti, W.; Bento, I.; Alexa, A.; Reményi, A., Co-regulation of the transcription controlling ATF2 phosphoswitch by JNK and p38. *Nat. Commun.* **2020,** *11*, 5769.
5. Petzold, G.; Fischer, E. S.; Thomä, N. H., Structural basis of lenalidomide-induced CK1α degradation by the CRL4CRBN ubiquitin ligase. *Nature* **2016,** *532*, 127-130.
6. Angell, R. M.; Angell, T. D.; Bamborough, P.; Bamford, M. J.; Chung, C.-w.; Cockerill, S. G.; Flack, S. S.; Jones, K. L.; Laine, D. I.; Longstaff, T.; Ludbrook, S.; Pearson, R.; Smith, K. J.; Smee, P. A.; Somers, D. O.; Walker, A. L., Biphenyl amide p38 kinase inhibitors 4: DFG-in and DFG-out binding modes. *Bioorg. Med. Chem. Lett.* **2008,** *18*, 4433-4437.
